# Bridging macro bibliometrics and micro molecular mechanisms: an integrated analysis of global aortic dissection research

**DOI:** 10.3389/fcvm.2026.1822129

**Published:** 2026-08-31

**Authors:** Yunnan Hu, Yixie He, Fan Xu, Liangwan Chen

**Affiliations:** 1 Department of Cardiovascular Surgery, Fujian Medical University Union Hospital, Fuzhou, China; 2 First Clinical Medical School, Southern Medical University, Guangzhou, China

**Keywords:** aortic dissection, bibliometrics, endovascular therapy, immune microenvironment, machine learning, single-cell transcriptomics

## Abstract

**Background:**

Aortic dissection (AD) is a catastrophic cardiovascular emergency with high morbidity and mortality. Despite advances in diagnosis and treatment, its complex pathogenesis, optimal therapies, and postoperative complications remain major challenges. Although AD research has expanded substantially, quantitative analyses integrating global knowledge structures with molecular mechanisms remain limited. This study mapped the development, contributors, hotspots, and emerging frontiers of global AD research to support future investigation and clinical translation.

**Methods:**

AD-related literature published from January 1, 2000, to December 31, 2025, was retrieved from the Web of Science Core Collection and Scopus. Excel, VOSviewer, CiteSpace, Scimago Graphica, and bibliometrix were used for bibliometric analyses, and a logistic growth model characterized publication trends. Single-cell RNA sequencing datasets GSE254132 and GSE213740 served as discovery and external-validation cohorts, respectively. Seurat, Harmony, donor-level pseudobulk differential expression, and exact donor-label permutation tests were applied. Differentially expressed genes were linked to target-specific PubMed trajectories to distinguish established AD genes from literature-sparse candidates.

**Results:**

A total of 5,170 publications were included. Output accelerated after 2010 and reached 712 articles in 2025. China produced the most publications, whereas the United States had the highest citation count and strongest international collaboration. Four convergent frontiers emerged: endovascular therapy, inflammation and vascular-wall remodeling, candidate molecular markers, and machine-learning-based prognosis. In GSE254132, AD was associated with vascular smooth muscle cell remodeling and a higher proportion of SPP1⁺ remodeling macrophages. GSE213740 reproduced the direction of this remodeling program and showed a smaller increase in SPP1⁺ macrophages despite compositional differences. During 2000–2025, PubMed indexed 1,483 publications on angiopoietin-like 4 (ANGPTL4) and 240 on matrix metalloproteinase 19 (MMP19) across biomedical fields. Output increased by 49.9% and 43.5%, respectively, in 2021–2025 versus 2016–2020. Record-level screening identified only two original AD-relevant publications for each target.

**Conclusion:**

This study maps the global structure and changing priorities of AD research and uses human single-cell data as a biological cross-check and candidate-prioritization layer. The combined evidence identifies ANGPTL4 and MMP19 as literature-sparse molecules that warrant mechanistic investigation, not as validated therapeutic targets. Independent cohorts and functional experiments are required before either molecule can be assigned diagnostic or therapeutic value.

## 1 Introduction

AD is a life-threatening acute aortic disease pathologically characterized by intimal tear of the aorta, with blood flowing into the medial layer to form a false lumen. It features acute onset, rapid progression, and high mortality, and is widely recognized as one of the most challenging cardiovascular emergencies (1). According to the Stanford classification, type A dissection involves the ascending aorta and requires emergency surgical intervention, while type B dissection is confined to the descending aorta, for which medical therapy or interventional treatment can be selected based on the patient's condition (2). In recent years, with the intensification of population aging and advances in imaging diagnostic technology, the global incidence of AD has shown a continuous upward trend. Although age-standardized in-hospital mortality has declined in some countries (e.g., the United States and Canada) (3), its overall prognosis remains suboptimal. Prominent challenges persist, including difficulties in early identification, complex pathological mechanisms, controversies over treatment strategies, and an imperfect prevention and control system for postoperative complications (4). The discovery of novel biomarkers, the application of four-dimensional imaging technology, and the popularization of minimally invasive interventional approaches such as thoracic endovascular aortic repair (TEVAR) have provided new directions for the clinical management of AD. However, the translation efficiency of basic research into clinical practice is still low, and there is an urgent need to systematically sort out the research context of the field to identify key breakthrough paths for future development.

To address these challenges, scholars worldwide have conducted a large number of original studies on AD, focusing on epidemiological characteristics, genetic susceptibility mechanisms (e.g., exploration of microRNA regulatory networks), optimization of imaging diagnosis, comparison of surgical and interventional treatment strategies, prediction of postoperative complications, and long-term prognosis evaluation (5, 6). Over the past two decades, the number of relevant publications has maintained a sustained and rapid growth trend, research themes have become increasingly diversified, and interdisciplinary integration has deepened. However, traditional systematic reviews and meta-analyses, while effective in integrating evidence-based evidence for specific clinical questions, struggle to dynamically capture the macro-level evolutionary trajectory of research and the evolution of frontier hotspots across time periods and disciplines. Faced with an increasingly complex literature system, researchers are in urgent need of a quantifiable and visualizable analytical tool to objectively reveal the evolutionary laws of the disciplinary knowledge structure and provide a scientific basis for accurately grasping the development direction of the field.

Bibliometrics, as an important method for analyzing disciplinary development trends, can systematically present the migration path of research hotspots and the evolutionary logic of knowledge foundations through quantitative evaluation of indicators such as publication output characteristics, author and institutional collaboration networks, keyword co-occurrence maps, and literature co-citation relationships, combined with visualization analysis technologies such as VOSviewer and CiteSpace (7). At present, this method has been applied to research in multiple subfields of aortic diseases, such as global trend analysis of acute type A aortic dissection (2), tracking of hotspot evolution in type B dissection research (8), and frontier identification of abdominal aortic aneurysms (9). However, existing studies mostly focus on a single subtype or specific theme, lacking a long-cycle, panoramic bibliometric integrated analysis of the entire AD research field.

This study therefore combined a long-cycle analysis of WoSCC and Scopus records with a logistic publication model and human single-cell transcriptomics. The single-cell component was incorporated for two linked purposes: to test whether mechanistic themes emerging from the bibliometric maps were observable in diseased human aortic tissue, and to use donor-level differential expression to prioritize genes for a target-specific literature-gap assessment. Genes with reproducible disease-associated expression but little AD-specific literature were treated as hypotheses for future work, whereas well-studied genes provided an internal calibration of the approach. This design links macro-level knowledge structure to tissue-level evidence without claiming that literature scarcity proves novelty or that differential expression establishes therapeutic efficacy.

## 2 Methods

### 2.1 Data source and search strategy

**Figure 1:**
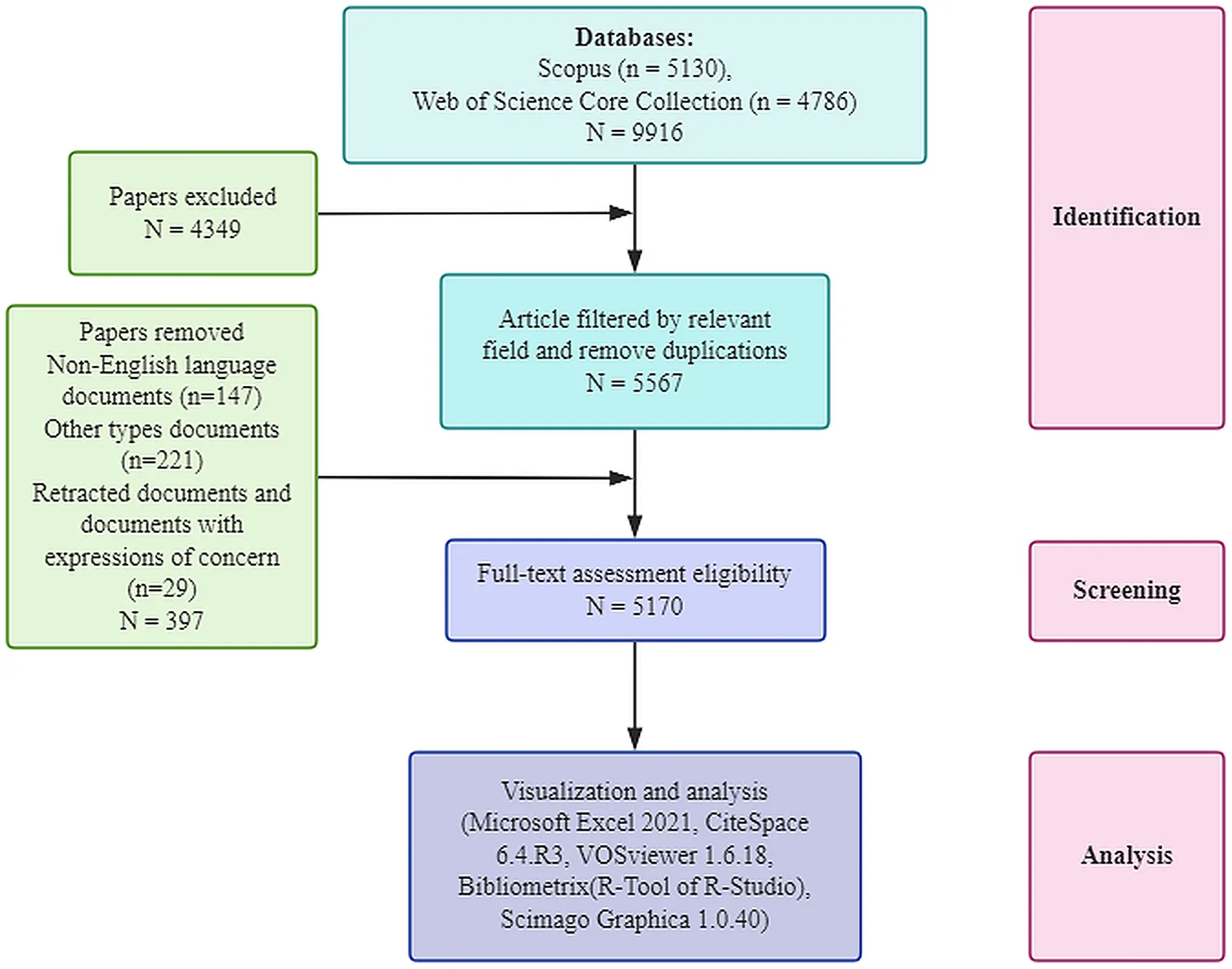
Flowchart of literature retrieval and screening. This flowchart illustrates the complete process of this study: retrieving literature related to aortic dissection from WoSCC and Scopus databases, performing deduplication and multiple rounds of standardized screening, ultimately including 5,170 articles, and conducting subsequent bibliometric analysis.

In this study, all bibliographic data were retrieved from WoSCC and Scopus databases (10), the validity of both of which has been well-documented in numerous prior bibliometric analyses. The time frame for the bibliometric analysis in this research was set from January 1, 2000, to December 31, 2025. Specifically, the search strategy applied in WoSCC was as follows: AK = (“Aortic dissection*” OR “Dissecting aortic aneurysm*” OR “Dissecting aneurysm* of the aorta” OR “Acute aortic syndrome” OR “Aortic intramural hematoma*” OR “Penetrating aortic ulcer*”) AND TI = (“Aortic dissection*” OR “Dissecting aortic aneurysm*” OR “Dissecting aneurysm* of the aorta” OR “Acute aortic syndrome” OR “Aortic intramural hematoma*” OR “Penetrating aortic ulcer*”). The search strategy adopted in Scopus was as follows: (TITLE(”Aortic dissection*” OR “Dissecting aortic aneurysm*” OR “Dissecting aneurysm* of the aorta” OR “Acute aortic syndrome” OR “Aortic intramural hematoma*” OR “Penetrating aortic ulcer*”)) AND (KEY(”Aortic dissection*” OR “Dissecting aortic aneurysm*” OR “Dissecting aneurysm* of the aorta” OR “Acute aortic syndrome” OR “Aortic intramural hematoma*” OR “Penetrating aortic ulcer*”)). From the above search results, a total of 4,786 documents were obtained from WoSCC and 5,130 from Scopus. After removing 4,349 duplicate records, excluding 147 non-English documents, filtering out 221 non-research publications (excluding original research, reviews and conference proceedings), and further excluding 29 retracted publications and publications with expressions of concern, a final set of 5,170 relevant English publications was downloaded for subsequent analysis. The complete records of each publication included publication details, research categories, affiliated countries/institutions, authors/co-authors, journal information, references and keywords (Figure 1).

WoSCC and Scopus were selected because they provide complementary coverage and metadata structures. WoSCC supplies standardized cited-reference records and is directly compatible with CiteSpace-based co-citation and burst analyses, whereas Scopus provides broader journal and conference coverage across clinical and interdisciplinary research (10). Combining them reduced dependence on a single indexing system, and deduplication based on digital object identifiers (DOIs), titles, and authors limited double counting. The title-plus-keyword search was intentionally specific to AD; consequently, articles in which AD was mentioned only in the abstract or full text may have been missed. This trade-off and the residual effects of database coverage are considered in the limitations.

### 2.2 Bibliometric analysis and target-level publication trends

Microsoft Office Excel 2021, CiteSpace 6.4.R3 (11), VOSviewer 1.6.18 (12), bibliometrix in R (13), and Scimago Graphica 1.0.40 (14) were used for annual-output analysis, collaboration mapping, keyword co-occurrence, co-citation analysis, thematic evolution, and visualization. CiteSpace was used for temporal and co-citation structures, VOSviewer for network construction and clustering, and bibliometrix for productivity, collaboration, and theme-evolution indicators. For the target-gap analysis, the top 50 up-regulated macrophage genes from donor-pseudobulk differential expression were matched to AD-specific PubMed counts. To evaluate whether *ANGPTL4* and *MMP19* were both underexplored in AD and attracting broader interest, annual PubMed searches were performed for 2000–2025. General publication trajectories used title/abstract-fielded target queries: “*ANGPTL4*” OR “angiopoietin-like 4”, and “*MMP19*” OR “matrix metalloproteinase 19” OR “matrix metallopeptidase 19”. The AD searches used the author-specified unfielded Boolean strings: ((*ANGPTL4*) OR (angiopoietin-like 4)) AND ((aortic dissection) OR (aortic dissections) OR (dissecting aortic aneurysm)); and ((*MMP19*) OR (matrix metalloproteinase 19) OR (matrix metallopeptidase 19)) AND ((aortic dissection) OR (aortic dissections) OR (dissecting aortic aneurysm)). Searches were run on July 19, 2026; journal issue years after 2025 were excluded. Because PubMed automatic term mapping retrieved records centered on angiopoietin-like 8 (*ANGPTL8*) and a published *MMP19* corrigendum, hits were screened by PubMed identifier (PMID), and non-target records and non-independent corrections were excluded. Each retained PMID was counted once and assigned to its journal issue year, preventing electronic and print dates from being counted as separate publications. Annual counts and totals for 2016–2020 versus 2021–2025 were compared.

### 2.3 Single-Cell RNA sequencing analysis and external validation

The discovery analysis used the human AD dataset GSE254132 from the Gene Expression Omnibus (GEO); GSE213740—comprising six acute type A aortic dissection donors and three non-dissected controls—was independently reanalyzed as an external cohort (15, 16). Single-cell RNA sequencing data were processed using Seurat. Genes detected in fewer than three cells were excluded. Cells were retained if they had 200–6,000 detected genes (nFeature_RNA), 500–40,000 unique molecular identifier counts (nCount_RNA), a mitochondrial transcript proportion (percent.mt) of ≤10%, and a hemoglobin transcript proportion (percent.hb) of ≤5%. Doublets were identified and removed independently within each biological sample using scDblFinder. After quality control, gene expression data were normalized using LogNormalize with a scale factor of 10,000, and 2,000 highly variable genes were selected using the variance-stabilizing transformation method.

The data were scaled with percent.mt regressed out, followed by principal component analysis based on the highly variable genes. Harmony integration was performed using SampleID as the batch variable, with theta = 2, lambda = 1, and a maximum of 10 iterations. For the global analysis, the first 30 Harmony dimensions were used to construct the nearest-neighbor graph, Louvain clustering was performed at a resolution of 0.6, and uniform manifold approximation and projection (UMAP) was generated using 30 neighbors and a minimum distance of 0.3. Cell types were annotated according to canonical lineage markers. Cluster marker genes were identified using the Wilcoxon rank-sum test, with a minimum expression fraction of 20%, a log₂ fold-change threshold of 0.25, and a Benjamini–Hochberg-adjusted *P* value of <0.05.

Macrophages were subsequently extracted and independently reclustered using LogNormalize normalization, 2,000 highly variable genes, and Harmony correction. The first 20 Harmony dimensions and a clustering resolution of 0.5 were used to identify macrophage states. For differential expression, biological donors rather than individual cells were treated as the units of statistical inference. Raw RNA counts were aggregated by donor and analyzed using the robust quasi-likelihood pseudobulk framework implemented in edgeR, with trimmed mean of M-values normalization. Differentially expressed genes were defined by a Benjamini–Hochberg false discovery rate of <0.05 and an absolute log₂ fold change of ≥1. Within-state differential expression of remodeling macrophages included only donors with at least 20 cells assigned to this state. In GSE213740, differences in cellular composition and macrophage program scores were evaluated using exact two-sided label-permutation tests across all 84 possible allocations of six AD donors and three control donors. Harmony-corrected embeddings were used exclusively for clustering and visualization; all differential expression analyses used uncorrected raw counts.

## 3 Results

### 3.1 Publication and citation analysis

**Figure 2:**
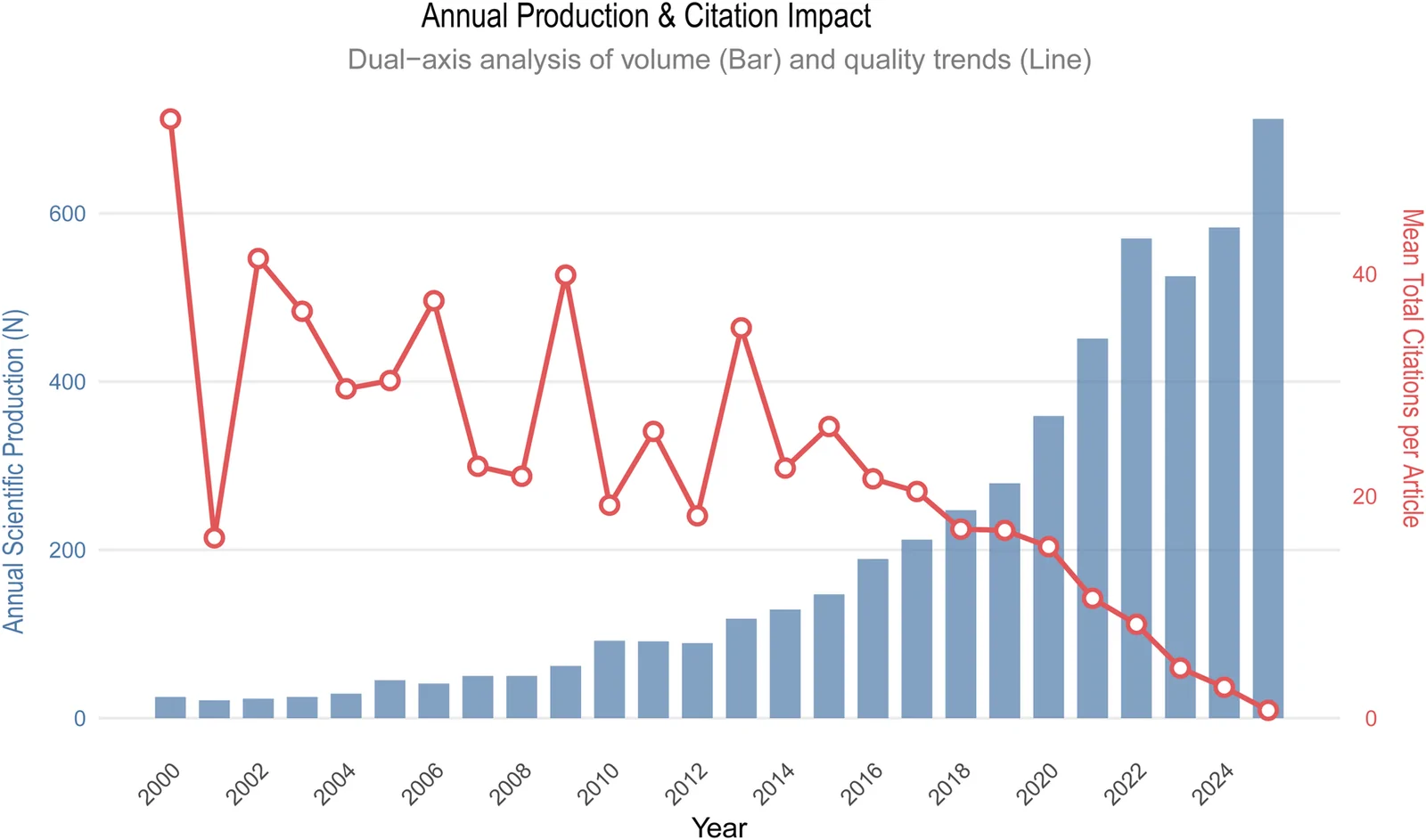
Annual publication volume and citation impact of aortic dissection research: a dual-axis analysis. This bar chart illustrates the trend in annual publication volume within the field of aortic dissection research from 2000 to 2025. A line graph concurrently displays the fluctuation characteristics of the average total citations per paper during the same period.

The period from 2000 to 2010 was the initial stage of development in the field of aortic dissection research, with the annual number of publications maintained at a low level of less than 100 articles and a gentle growth trend. After 2010, the field entered a period of rapid development, with the annual number of publications showing a sustained explosive growth, reaching 712 articles in 2025, an increase of 2748% compared with 2000. This fully reflects the continuously rising research heat and global academic attention in this field. Consistent with the growth trend of the number of publications, the total annual citation frequency in the field also showed a steady upward trend, from 0 in 2000, increasing year by year, and exceeding 10,000 times to 10,123 times in 2025, reflecting the continuous expansion of the academic influence and disciplinary recognition of research results in this field. It is worth noting that the average number of citations per article showed an overall fluctuating downward trend with the expansion of publication scale. It maintained a high level from 2000 to 2017, and then gradually declined. This phenomenon is consistent with the general law of bibliometrics: on the one hand, recently published literature still needs sufficient time to complete citation accumulation; on the other hand, the rapid expansion of publication volume in the field has diluted the average citation level of a single article to a certain extent (Figure 2).

**Figure 3:**
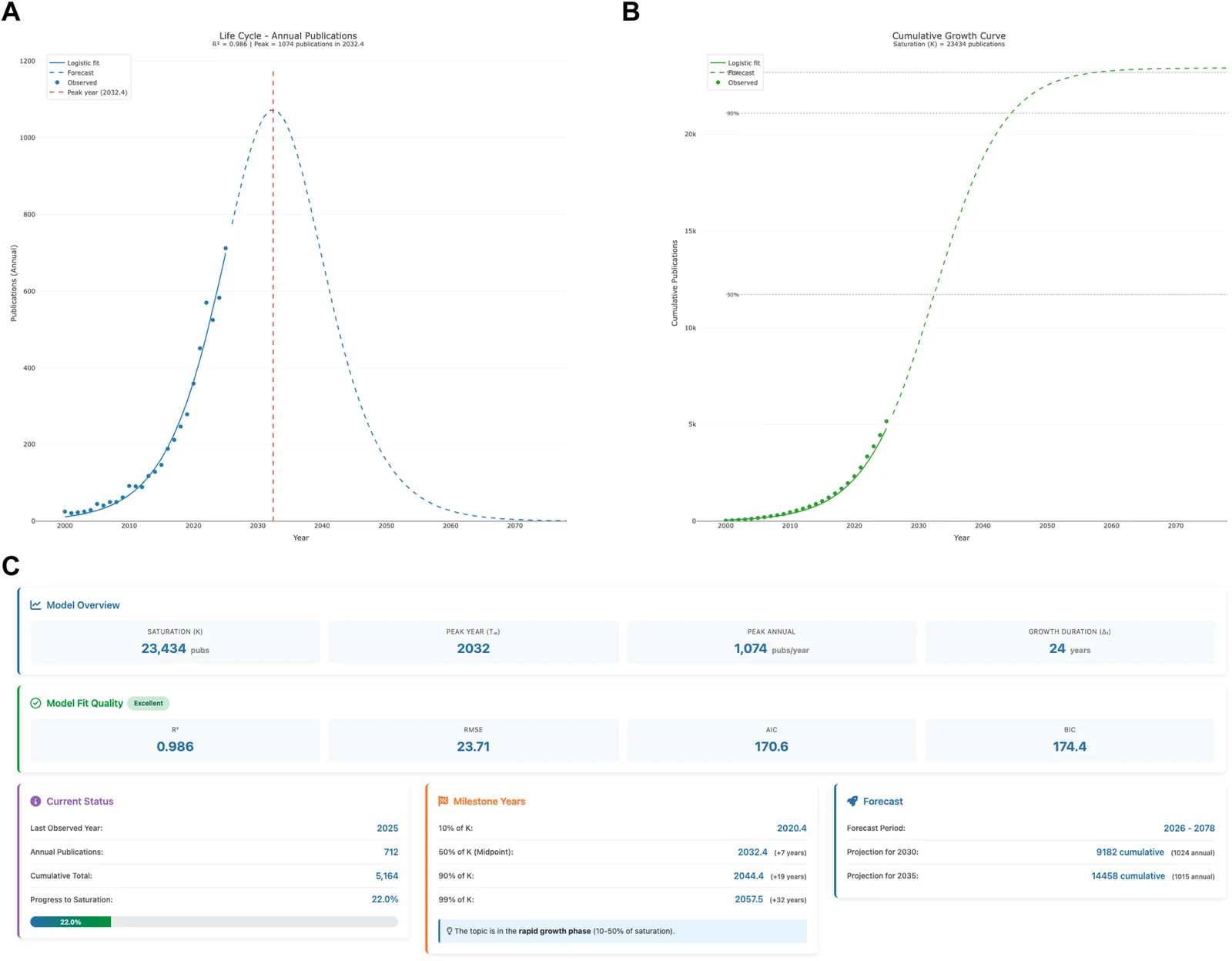
**(A)** Annual publication volume logistic growth curve fitting and trend forecast chart: completed logistic growth curve fitting and future trend forecasting for annual publication volume in aortic dissection research. The analysis confirms the peak publication volume will occur in 2032, with a model fitting goodness-of-fit *R*^2^ = 0.986. **(B)** Logistic Growth Curve Fitting Chart for Cumulative Publication Volume. Completed logistic growth curve fitting analysis for cumulative publication volume in aortic dissection research, determining the saturation value at 23,434 publications. **(C)** Summary Table of Logistic Growth Curve Model Parameters and Prediction Results: This table consolidates the core parameters of the logistic growth curve model, fitting quality, current state of field development, key milestone nodes, and projected future publication volume.

To further clarify the development stage and future evolutionary trend of the field, this study used a logistic growth curve model to fit and predict the annual publication data. The results showed that *R*^2^ = 0.986, the saturation value *K* of the number of publications in this field was 23,434 articles, and the peak annual publication volume will appear in 2032, with an estimated peak of 1,074 articles. As of 2025, the total cumulative number of publications in the field was 5,164 articles, only reaching 22.0% of the saturation value, which is in the rapid growth stage of the 10%–50% saturation interval, with extremely strong development vitality. From the perspective of development milestones, the field reached 10% of the saturation value in 2020, is expected to reach the development midpoint of 50% of the saturation value in 2032, 90% of the saturation value in 2044, and close to 99% of the saturation value in 2057. From the specific prediction results, the field is expected to have a cumulative 9,182 publications and 1,024 annual publications in 2030, and a cumulative 14,458 publications and 1,015 annual publications in 2035. The annual publication volume will gradually decline after reaching the peak in 2032, while the cumulative publication volume will continue to grow and gradually approach the saturation value. This indicates that the field is currently in the golden stage of rapid development, and still has sufficient academic exploration space and development potential in the future (Figures 3A–C).

### 3.2 Distribution of countries/regions

Country/region analysis in bibliometrics is used to evaluate the geographical distribution of scientific research output, quantify national contribution, and reveal international cooperation models, providing empirical basis for the formulation of regional scientific research policies and global influence analysis. Among the 98 countries with collected information, China ranked first in the world in terms of publication volume with an absolute advantage of 1,921 articles (37.16%), serving as the core publication entity in this field, followed by the United States (938 articles, 18.14%), Japan (818 articles, 15.82%), etc. From the perspective of academic influence, the United States ranked first in the world with 17,151 total citations from 938 publications, reflecting the high quality of its academic research results. China followed closely with 16,982 total citations, and together they constituted the first echelon of academic influence in this field (Table 1).

**Table 1:** Ranking of the top ten major countries/regions in the field of aortic dissection from 2000 to 2025.

Rank	Countries	Documents	Countries	Citations	Countries	Total link strength
1	China	1,921	USA	17,151	USA	606
2	USA	938	China	16,982	Italy	470
3	Japan	818	Germany	12,180	Germany	468
4	Germany	369	Japan	10,316	England	377
5	Italy	281	Italy	8,485	Spain	253
6	England	255	England	6,209	France	211
7	Taiwan	129	France	3,665	China	202
8	France	123	Switzerland	3,277	Finland	198
9	Canada	107	Spain	3,184	Belgium	177
10	Switzerland	101	Canada	2,690	Canada	146

**Figure 4:**
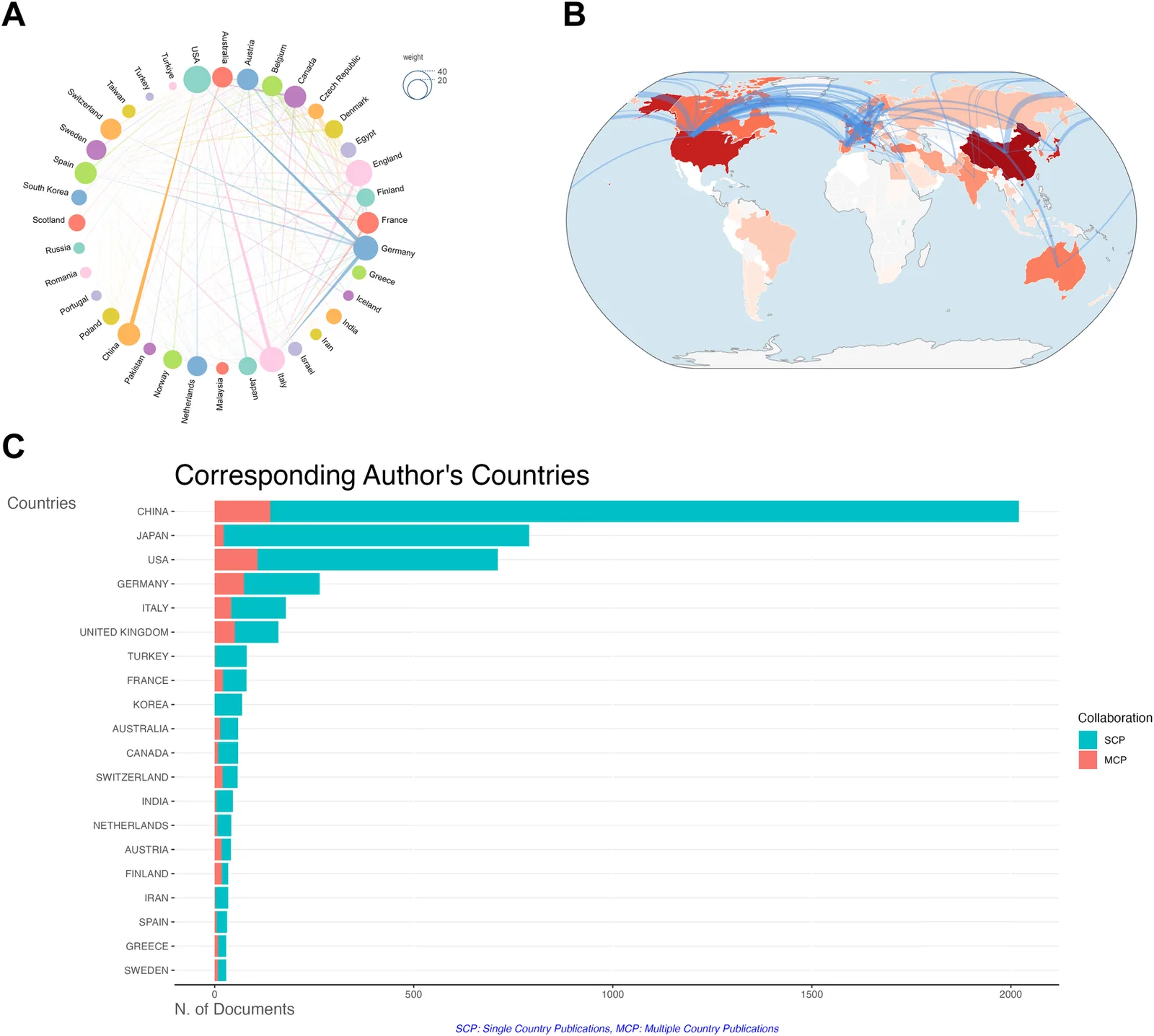
**(A)** Inter-country collaboration network map, where node size corresponds to a country's publication volume and line thickness represents the intensity of collaboration between countries, revealing the global cooperation landscape and core nodes in this field. **(B)** Global regional collaboration network spatial distribution map, visually presenting the geographic distribution characteristics of the international collaboration network in aortic dissection research, clearly showing that international cooperation in this field is primarily concentrated in three core regions: North America, Europe, and East Asia. **(C)** Stacked bar chart of publication collaboration patterns among core country corresponding authors. This chart statistically analyzes the publication collaboration patterns of core country corresponding authors in global aortic dissection research, displaying the distribution and proportion of SCPs versus MCPs across nations.

In terms of international cooperation, the inter-country cooperation network in the global aortic dissection research field presents an overall dense and interconnected characteristic, and a global cooperation system with the United States, Germany, Italy, and China as core nodes has been formed. Among them, the United States has a total link strength of 606, serving as the core academic hub for international cooperation in this field. Italy (470), Germany (468), and the United Kingdom (377) also have extremely high international cooperation activity, while China has a total link strength of 202, ranking 7th in the world, with considerable room for improvement in the breadth and depth of its international cooperation (Figure 4A). The global regional collaboration network shows that international cooperation links are mainly concentrated in the three core regions of North America, Europe, and East Asia. The cooperation links between European and American countries across the Atlantic Ocean and cross-regional cooperation links in Eurasia are the most intensive, forming a cooperation network covering major research countries around the world (Figure 4B). The analysis of corresponding author's country and publication cooperation models shows that the aortic dissection-related research of core countries around the world is dominated by single-country publications (SCPs), while multiple-country publications (MCPs) account for a certain proportion. Among them, the United States, Germany and other European and American countries have a relatively higher proportion of transnational cooperative publications, while China has a higher proportion of single-country publications (Figure 4C).

### 3.3 Distribution of institutions

By quantitatively evaluating the paper output, citation influence and cooperation network of 3,998 scientific research institutions participating in the research in this field, this section reveals the scientific research strength and competitive situation of institutions, and provides decision support for the allocation of scientific research resources, partner selection and science and technology policy formulation. Among the top 10 institutions in the world in terms of publication volume, 9 are Chinese institutions. Among them, Capital Medical University ranked first with 215 publications, far exceeding other institutions, followed by Fudan University (129 articles), Fujian Medical University (121 articles), etc. The University of Michigan in the United States (85 articles) is the only non-Chinese institution in the global top 10. Notably, it is also the most cited institution (4,069 citations), indicating that the research results of the University of Michigan in the field of aortic dissection have received extensive attention from the academic community (Table 2).

**Table 2:** Ranking of the top ten major institutions in the field of aortic dissection from 2000 to 2025.

Rank	Institution	Publications	Original country	Institution	Citations	Original country
1	Capital Medical University	215	China	University of Michigan	4,069	USA
2	Fudan University	129	China	Massachusetts General Hospital	2,734	USA
3	Fujian Medical University	121	China	Capital Medical University	2,660	China
4	Chinese Academy of Medical Sciences and Peking Union Medical College	117	China	Fudan University	1,863	China
5	Huazhong University of Science and Technology	115	China	University of Tokyo	1,794	Japan
6	Nanjing Medical University	101	China	University of Pennsylvania	1,629	USA
7	Nanjing University	92	China	Stanford University	1,590	USA
8	University of Michigan	85	USA	Chinese Academy of Medical Sciences	1,588	China
9	Central South University	82	China	Mayo Clinic	1,510	USA
10	Chinese Academy of Medical Sciences	70	China	University Hospital Bern	1,461	Switzerland

**Figure 5:**
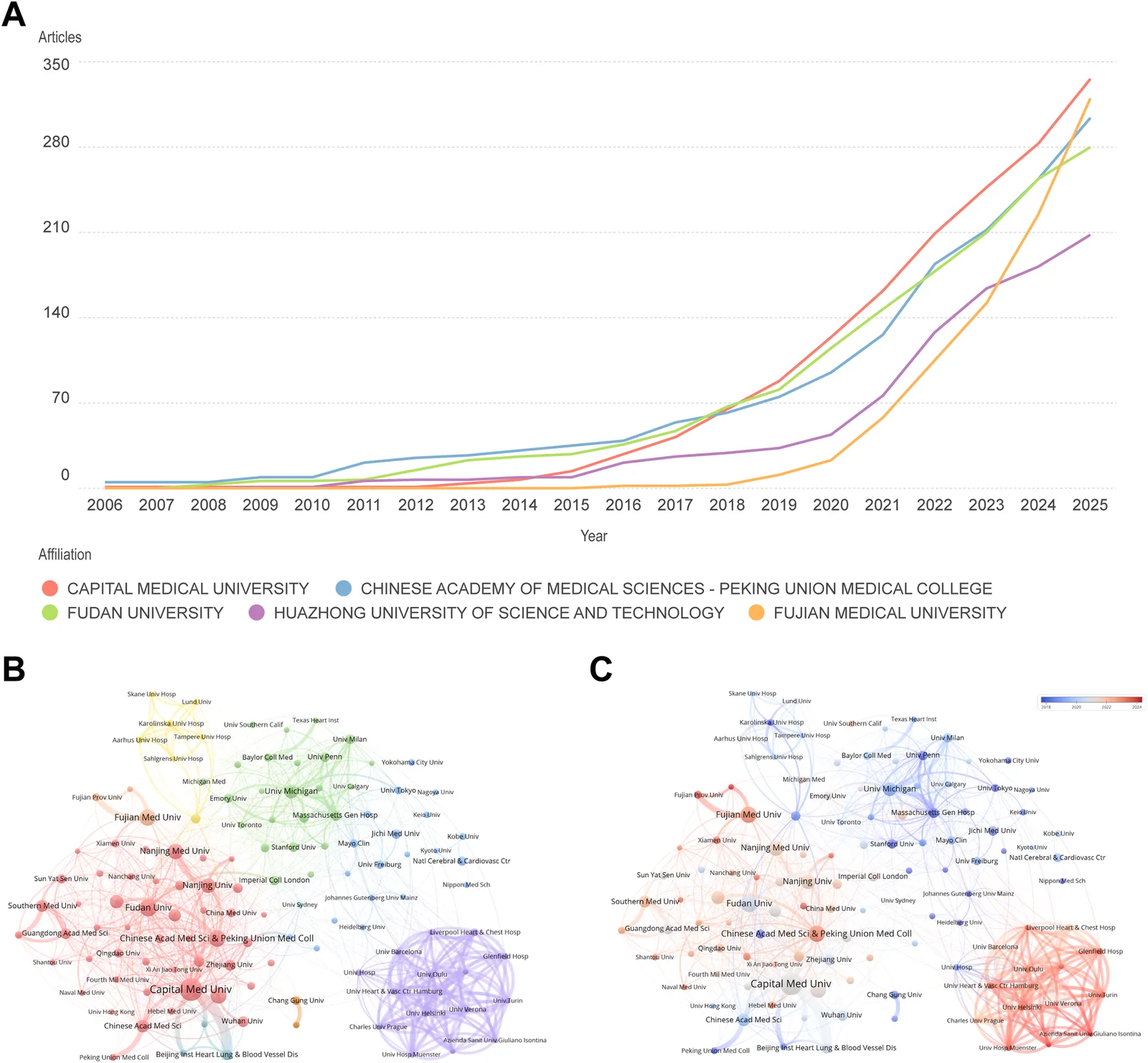
**(A)** Annual publication volume trend chart for core research institutions: this chart illustrates the cumulative annual publication volume changes in aortic dissection research from 2006 to 2025 across six domestic core institutions. **(B)** Global Research Institution Collaboration Network Map: Node size corresponds to total publication volume, while line thickness represents collaboration intensity between institutions. This reveals the distribution pattern of three major geographically clustered collaboration clusters: China, North America, and Europe. **(C)** Time evolution map of institutional collaboration networks, which uses color gradient to map the year of collaboration occurrence, visualizes the temporal evolution law of institutional collaboration networks in global aortic dissection research, and reveals the characteristic of significantly increased collaboration activity of the European research cluster in recent years.

From the perspective of time trend, the publication volume of core Chinese institutions such as Capital Medical University, Peking Union Medical College & Chinese Academy of Medical Sciences, Fudan University, Huazhong University of Science and Technology, and Fujian Medical University has shown a sustained and rapid growth trend since 2015, especially with a significant increase after 2020, fully demonstrating China's research vitality and development potential in this field (Figure 5A). The institutional cooperation relationship is shown in Figure 5B. The institutional cooperation of global aortic dissection research presents obvious geographical agglomeration characteristics, and three core clusters have been formed: the Chinese cluster with Capital Medical University, Peking Union Medical College & Chinese Academy of Medical Sciences, Fudan University, Nanjing Medical University, etc. as the core; the North American cluster with the University of Michigan, Massachusetts General Hospital, Stanford University, Mayo Clinic, etc. as the core; and the European cluster with Liverpool Heart and Chest Hospital, Helsinki University Hospital, University of Verona, etc. as the core. The cooperation network among Chinese institutions is the densest, forming a close internal cooperation system, while European and American institutions show more characteristics of cross-regional cooperation. From the evolution of cooperation time, the cooperation activity of the European cluster has increased significantly in recent years (Figure 5C).

### 3.4 Distribution of authors and co-cited authors

Author and co-cited author analysis helps to identify high-yield core scholars, evaluate individual scientific research output, analyze and reveal the knowledge association and academic network among scholars, and identify research schools (17). Among the top 10 authors in the world in terms of publication volume, 7 are Chinese scholars. Among them, Chen Liangwan ranked first with 92 publications, followed by Sun Lizhong (63 articles), Zhang Hongjia (59 articles), Wang Dongjin (57 articles), etc. In terms of author citation frequency, Nienaber, Christoph A. became the most concerned author with 2,003 co-citations. From a global perspective, he has published 1,333 cumulative articles, with a total of 49,221 citations and an h-index of 100. His research focuses on core fields such as the diagnosis and treatment of aortic diseases, with top academic influence. From the in-field perspective of this study, he has published 45 articles in the field of aortic dissection, with a total of 5,111 citations and a local h-index of 26. His research keywords are highly focused on “Aortic Dissection”, making him a key leader in this field (Table 3, Supplementary Figure S1).

**Table 3:** Ranking of the top ten major authors in the field of aortic dissection from 2000 to 2025.

Rank	Author	Documents	Citations	Countries/regions	Institution	Author	co-citations	Total link strength	Countries/regions	Institution
1	Chen, Liangwan	92	472	China	Fujian Medical University	Nienaber, Ca	2,003	19,396	England	Royal Brompton and Harefield NHS Foundation Trust
2	Sun, Lizhong	63	1,173	China	Capital Medical University	Erbel, R	1,083	10,090	Germany	University of Duisburg Essen
3	Zhang, Hongjia	59	635	China	Capital Medical University	Evangelista, A	985	10,800	Spain	Teknon Medical Center
4	Wang, Dongjin	57	439	China	Nanjing University	Hagan, Pg	939	8,176	USA	University of Michigan
5	Patel, Himanshu J.	45	1,564	USA	University of Michigan	Tsai, Tt	840	10,047	USA	Bruce W. Carter Department of Veterans Affairs Medical Center
6	Zhu, Junming	44	832	China	Capital Medical University	Trimarchi, S	682	9,296	Italy	IRCCS Ca’ Granda Maggiore Policlinic Hospital of Milan
7	Czerny, Martin	42	3,056	Germany	University of Freiburg	Rylski, B	679	7,611	Germany	Robert Bosch Hospital
8	Yang, Bo	42	777	USA	University of Michigan	Czerny, M	584	6,098	Germany	University of Freiburg
9	Fu, Weiguo	41	506	China	Fudan University	Suzuki, T	555	6,391	England	University of Leicester
10	Nienaber, Christoph A.	38	4,219	England	Royal Brompton and Harefield NHS Foundation Trust	Pape, La	527	5,366	Italy	Antonio Cardarelli Hospital

**Figure 6:**
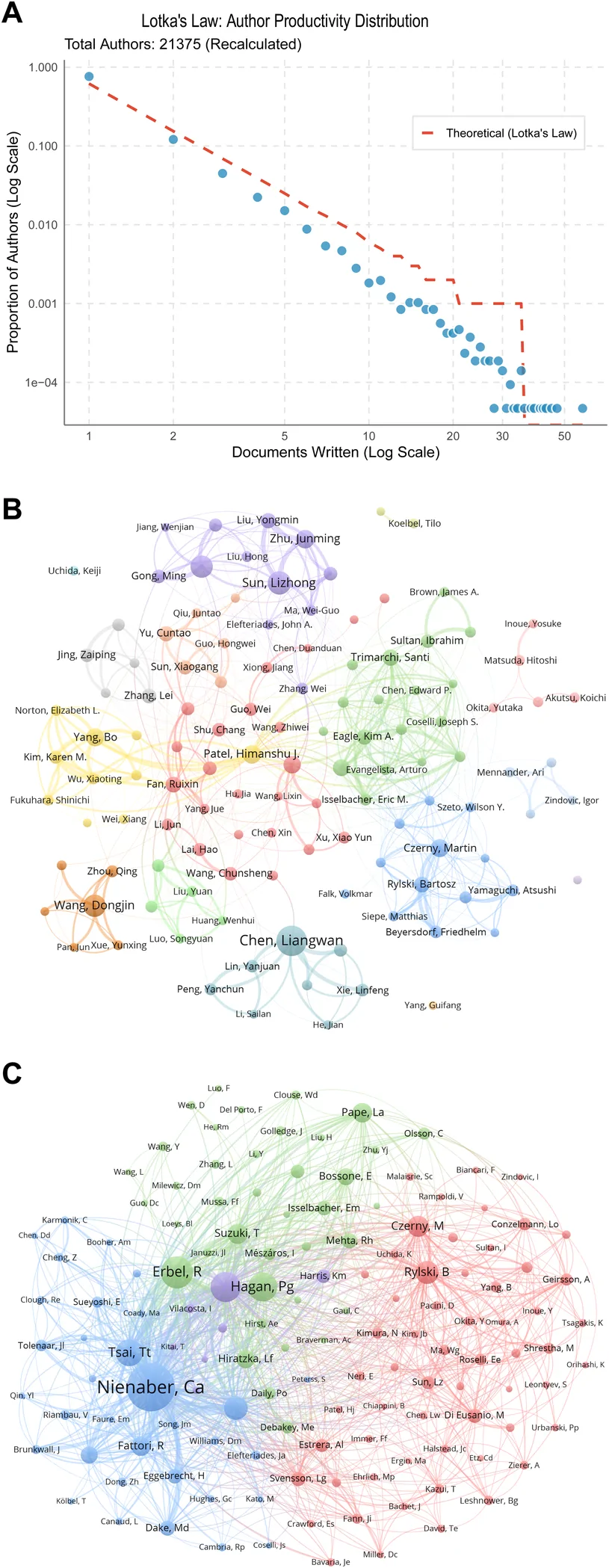
**(A)** Author productivity distribution map based on lotka's Law, which verifies that the publication productivity distribution of 21,375 authors in the global field of aortic dissection research is highly consistent with the theoretical curve of lotka's Law, presenting the distribution characteristic that a small number of high-yield authors contribute the main research results in the field. **(B)** Author collaboration network map in the field of aortic dissection research, where the node size corresponds to the publication volume of each author, and the line thickness represents the strength of collaboration between authors, presenting the characteristics of two major geographically aggregated collaboration clusters centered on core Chinese and European scholars respectively. **(C)** Author co-citation network map in the field of aortic dissection research, where the node size corresponds to the co-citation frequency of each author, clearly presenting the co-citation network structure with top European and American scholars as the core and the clustering characteristics of different research directions in this field.

The fitting results of Lotka's Law showed that the publication productivity distribution of 21,375 authors worldwide is highly consistent with the theoretical curve. The vast majority of authors published only 1 article, while a small number of high-yield authors contributed the main research results in the field, which is in line with the general law of bibliometrics (Figure 6A). The cooperative relationship between authors is visualized by VOSviewer, as shown in Figure 6B. The author cooperative relationship in this field presents obvious geographical agglomeration characteristics, and an East Asian cooperation cluster centered on core Chinese scholars such as Chen Liangwan, Sun Lizhong, and Zhu Junming, and a European cooperation cluster centered on German scholars such as Czerny, Martin and Rylski, Bartosz have been formed. The author co-citation network shows that the co-citation core of global aortic dissection research is mainly composed of top European and American scholars. Scholars such as Nienaber, Ca, Erbel, R, Hagan, Pg, and Tsai, Tt are in the central position of the co-citation network, serving as key nodes for academic inheritance and knowledge dissemination in this field. Among them, the blue cluster with Nienaber, Ca as the core focuses on the diagnosis and clinical management of aortic dissection; the green cluster with Erbel, R and Hagan, Pg as the core focuses on the epidemiology and prognosis research of aortic diseases; the red cluster with Czerny, M and Rylski, B as the core represents the direction of surgical and interventional treatment of aortic dissection (Figure 6C).

### 3.5 Journal publication analysis

**Figure 7:**
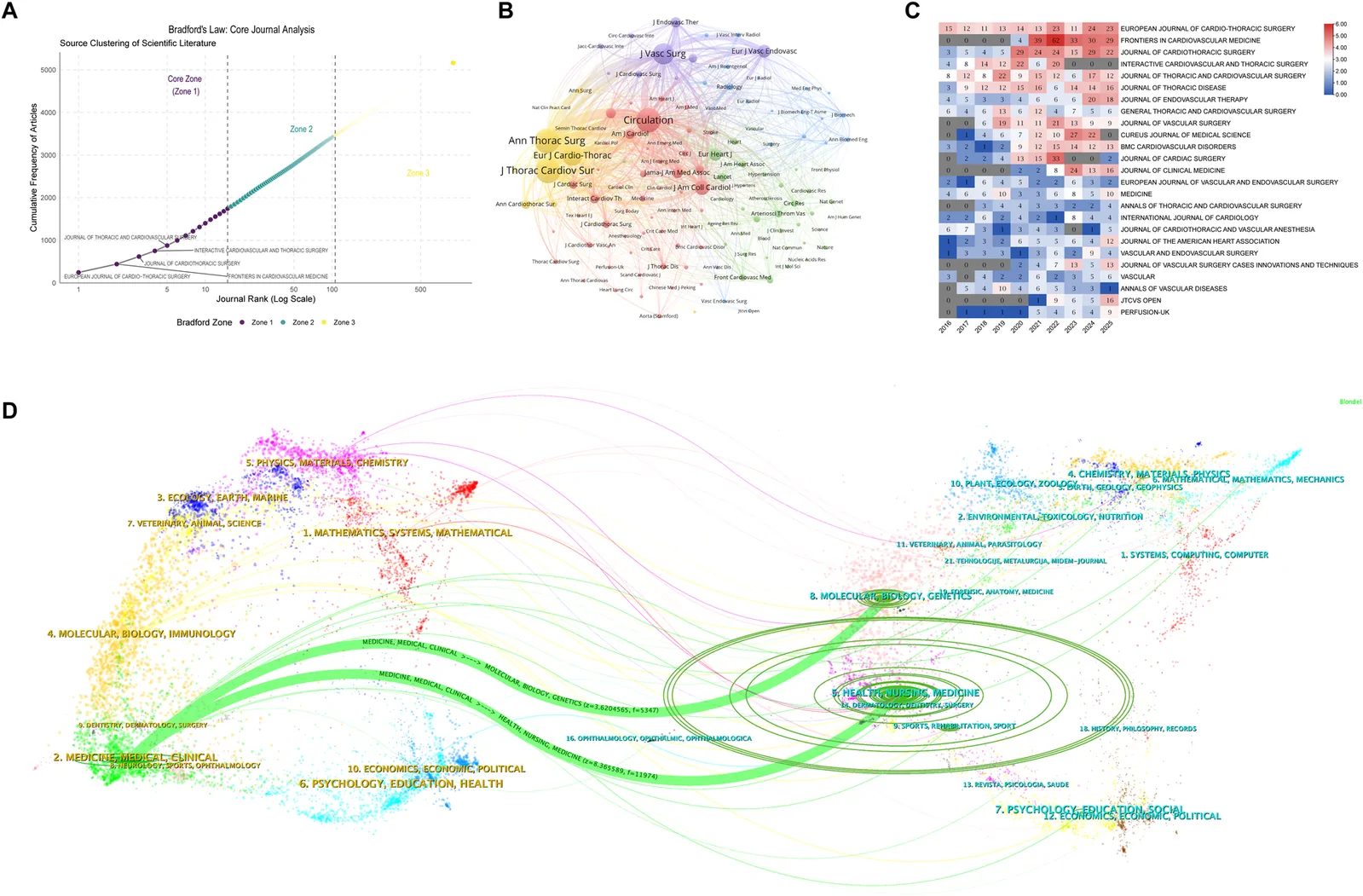
**(A)** Core journal zoning analysis map based on bradford's Law, which divides journals in the field of aortic dissection research into the core zone, intermediate zone and marginal zone, and clarifies the dominant position of core zone journals in the publication contribution of the field. **(B)** Journal co-citation network map, where the node size corresponds to the co-citation frequency of each journal, clearly presenting the clustering characteristics of different research directions and knowledge association relationships among journals in the field. **(C)** Time evolution heat map of annual publication volume of core journals, which uses color gradient to map the volume of publications, and shows the changing trend and temporal distribution characteristics of annual publication scale of core journals in the field from 2016 to 2025. **(D)** Dual-map overlay plot of journal disciplines, which visualizes the cross-disciplinary citation relationships and interdisciplinary research pattern in the field of aortic dissection research through citation flow.

Bibliometric analysis was conducted on 7,166 journals participating in the research and publication in this field, to more comprehensively identify core journals and important publication platforms in the field, and reveal the disciplinary knowledge dissemination channels and the evolution of research hotspots. The top 10 journals in terms of publication volume are mainly cardiothoracic and vascular surgery specialist journals. *European Journal of Cardio-Thoracic Surgery* ranked first with 244 publications, serving as the core publication carrier in this field, followed by *Frontiers in Cardiovascular Medicine* and *Journal of Cardiothoracic Surgery*, with 197 and 176 publications respectively. These journals cover Journal Citation Reports (JCR) Q1 to Q3 quartiles, providing diversified publication channels for research results in the field of aortic dissection. In terms of co-citation, *Annals of Thoracic Surgery*, *Journal of Thoracic and Cardiovascular Surgery*, and *Circulation* ranked the top three with total citations of 9,177, 9,006, and 8,848 times respectively. Eight of the top 10 co-cited journals are in the JCR Q1 quartile, with a higher overall impact factor level, serving as the core carrier for knowledge inheritance and academic influence dissemination in this field (Table 4). The core journal zoning results based on Bradford's Law show that the distribution of journal publications in this field can be divided into the core zone (Zone 1), the intermediate zone (Zone 2) and the marginal zone (Zone 3). The core zone has the smallest number of journals, but contributes the vast majority of publications in the field. Journals with leading publication volume such as *European Journal of Cardio-Thoracic Surgery* and *Journal of Thoracic and Cardiovascular Surgery* are all in the core zone, further confirming their core academic status in this field (Figure 7A).

**Table 4:** Ranking of the top ten major journals in the field of aortic dissection from 2000 to 2025.

Rank	Journal	Publications	2024 impact factor	JCR quartile	Co-cited journal	Citations	2024 impact factor	JCR quartile
1	European Journal of Cardio-Thoracic Surgery	244	3.0	Q2	Annals of Thoracic Surgery	9,177	3.9	Q1
2	Frontiers in Cardiovascular Medicine	197	2.9	Q2	Journal of Thoracic and Cardiovascular Surgery	9,006	4.4	Q1
3	Journal of Cardiothoracic Surgery	176	1.5	Q3	Circulation	8,848	38.7	Q1
4	Interactive Cardiovascular and Thoracic Surgery	133	2.1	Q3	Journal of Vascular Surgery	6,591	3.6	Q1
5	Journal of Thoracic and Cardiovascular Surgery	126	4.4	Q1	European Journal of Cardio-Thoracic Surgery	5,958	3.0	Q2
6	Journal of Thoracic Disease	121	1.9	Q3	Journal of the American College of Cardiology	3,293	22.3	Q1
7	Journal of Endovascular Therapy	111	1.5	Q3	European Journal of Vascular and Endovascular Surgery	2,525	6.8	Q1
8	General Thoracic and Cardiovascular Surgery	103	1.3	Q3	European Heart Journal	2,380	35.7	Q1
9	Journal of Vascular Surgery	99	3.6	Q1	JAMA: Journal of the American Medical Association	1,679	55.0	Q1
10	Cureus Journal of Medical Science	89	1.3	Q2	American Journal of Cardiology	1,671	2.1	Q3

The journal co-citation network presents clear clustering characteristics of research directions. Among them, the yellow cluster centered on *Annals of Thoracic Surgery* and *Journal of Thoracic and Cardiovascular Surgery* focuses on open surgery and clinical research of cardiothoracic surgery for aortic dissection; the red cluster centered on *Circulation* and *Journal of the American College of Cardiology* focuses on evidence-based medicine and basic clinical research in cardiovascular medicine; the purple cluster centered on *Journal of Vascular Surgery* and *European Journal of Vascular and Endovascular Surgery* revolves around endovascular interventional therapy and imaging evaluation of aortic dissection; the green cluster centered on *European Heart Journal* and *Circulation Research* focuses on basic medicine and molecular mechanism exploration of aortic diseases (Figure 7B).

The time evolution heat map of the publication volume of core journals shows that the overall publication scale of core journals in this field is increasing year by year. The publication volume of *Frontiers in Cardiovascular Medicine* increased significantly from 2021 to 2023, while surgical specialist journals such as *European Journal of Cardio-Thoracic Surgery* and *Journal of Cardiothoracic Surgery* have maintained a stable publication scale for a long time, providing a stable academic output platform for the field (Figure 7C). Figure 7D adopts the dual mapping overlay technology of journal disciplines, and intuitively presents the inter-disciplinary citation relationship and interdisciplinary research pattern in the field of aortic dissection research through the citation flow from the citing disciplines on the left to the cited disciplines on the right via connecting lines. The significant green lines in the figure indicate that the research results of journals focusing on health science, nursing medicine, molecular science, biology and genetics are frequently cited by journals related to medicine, medical treatment and clinical practice.

### 3.6 Keyword analysis

**Figure 8:**
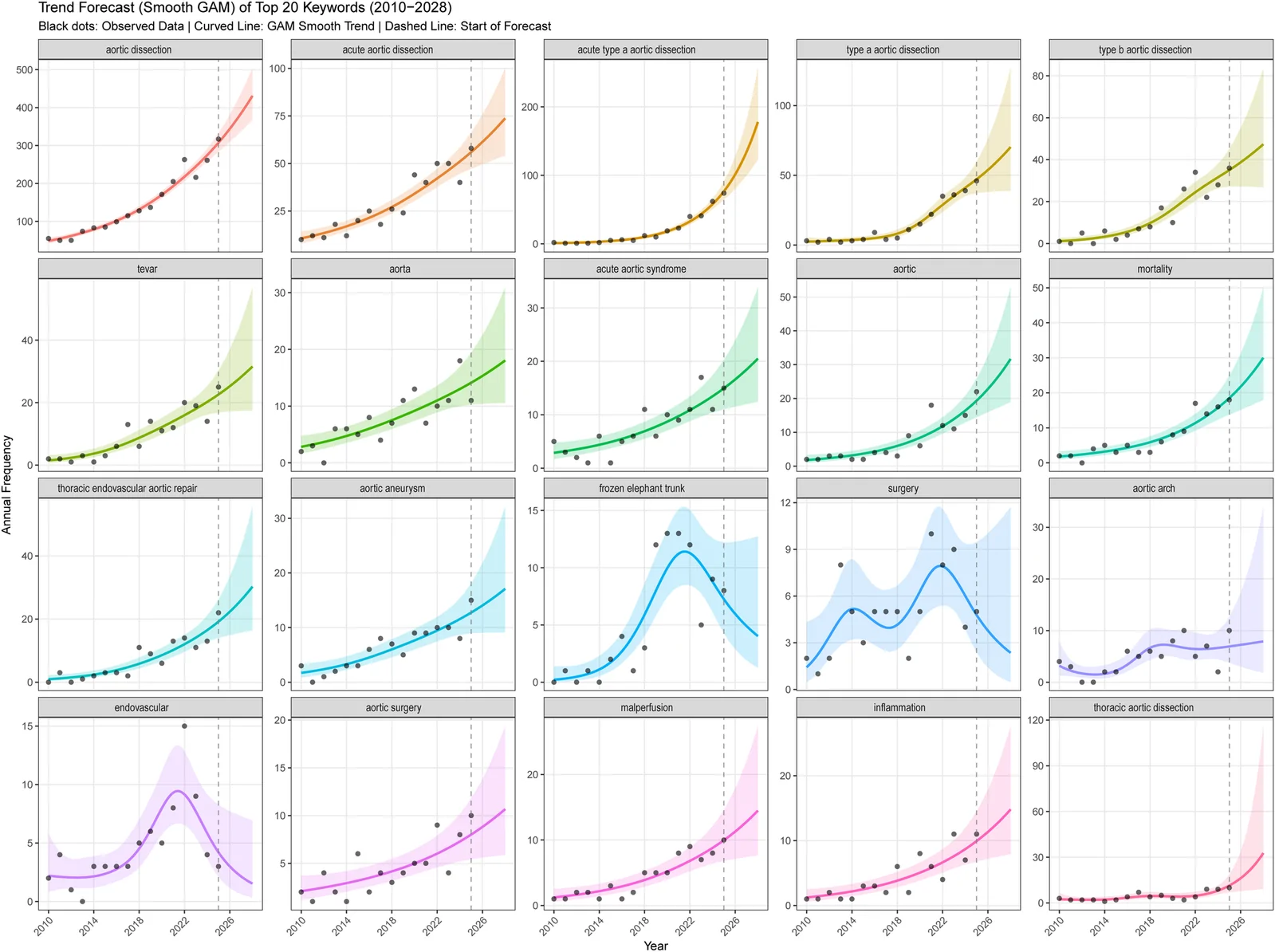
This figure shows the observed data of annual occurrence frequency of keywords from 2010 to 2025 with black dots, completes data fitting and popularity trend prediction from 2025 to 2028 with a smooth curve, and presents the popularity evolution law and future development trend of different research topics.

Keyword analysis, as the core method of bibliometrics, plays a key role in revealing the disciplinary research pattern and predicting its development trend. In the research of this field, “aortic dissection” is the most core basic keyword in this field, appearing 2,759 times; “type a aortic dissection” (856 times), “acute aortic dissection” (547 times), “tevar” (486 times), etc. are high-frequency core keywords (Table 5). The annual frequency trend prediction of the top 20 keywords from 2010 to 2028 based on the generalized additive model (GAM) is shown in Figure 8. The popularity of keywords related to disease classification and endovascular interventional therapy such as “aortic dissection”, “type a/b aortic dissection”, and “tevar” continues to rise, and is predicted to maintain rapid growth before 2028, serving as the core hotspots in the field; themes related to mechanism and prognosis such as “inflammation”, “malperfusion”, and “mortality” also show a continuous upward trend, becoming the key research directions in the future; while the popularity of traditional surgery-related keywords such as “frozen elephant trunk” and “surgery” has passed the peak, and is predicted to continue to decline in the follow-up (Figure 8).

**Table 5:** Ranking of the top twenty major keywords in the field of aortic dissection from 2000 to 2025.

Rank	Keyword	Occurrences	Total link strength	Rank	Keyword	Occurrences	Total link strength
1	aortic dissection	2,759	3,286	11	outcome	132	288
2	type a aortic dissection	856	955	12	aortic aneurysm	120	214
3	acute aortic dissection	547	553	13	surgery	114	240
4	tevar	486	958	14	stent graft	109	318
5	type b aortic dissection	409	687	15	case report	105	176
6	mortality	176	354	16	aortic remodeling	104	230
7	risk factor	167	305	17	aortic surgery	104	200
8	acute aortic syndrome	157	323	18	malperfusion	104	223
9	aorta	157	374	19	thoracic aortic dissection	104	97
10	frozen elephant trunk	138	268	20	computed tomography	96	202

**Figure 9:**
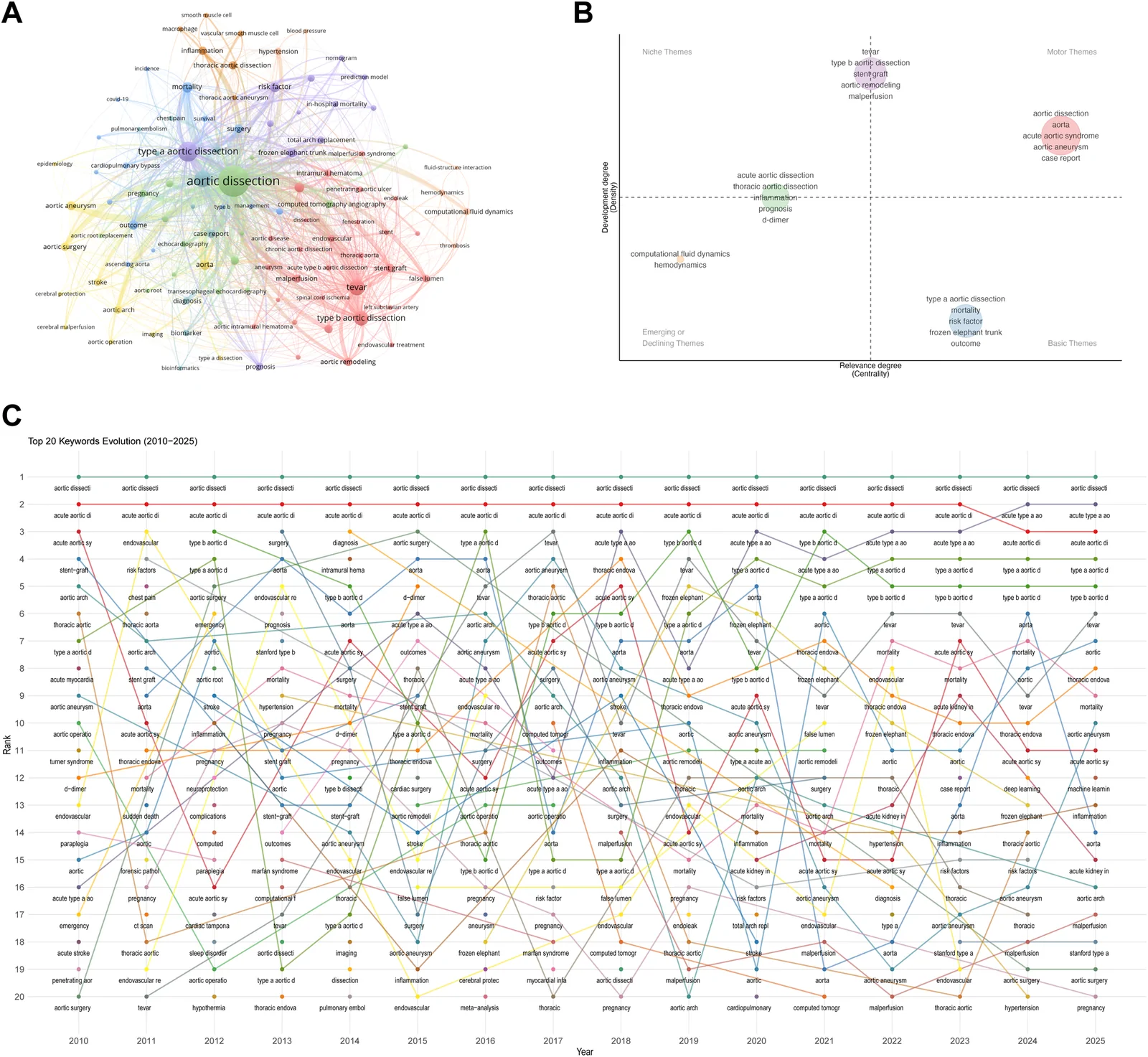
**(A)** Keyword co-occurrence network clustering map, where the node size corresponds to the occurrence frequency of keywords, and the line thickness represents the association strength between keywords, clearly presenting the clustering characteristics of core research topics and knowledge association relationships in this field. **(B)** Four-quadrant distribution map of keyword themes, which takes centrality as the horizontal axis and development degree as the vertical axis to divide research topics into four categories, and clarifies the development status and academic status in the field of different research topics. **(C)** Time evolution map of top 20 keywords from 2010 to 2025, which shows the annual ranking changes of high-frequency keywords in the field from 2010 to 2025, and reveals the temporal migration law of research hotspots and theme evolution trend in this field.

Based on the VOSviewer tool, visual cluster analysis of the keyword co-occurrence network was carried out through link strength, and the results are shown in Figure 9A: the green cluster, centered on the core keyword “aortic dissection”, is the research cluster of disease basic definition, clinical classification and pathological phenotype, which constitutes the core research framework of the entire field; the purple cluster, centered on “type a aortic dissection”, focuses on open surgical techniques and perioperative management research of type A aortic dissection; the red cluster, revolving around “tevar” and “type b aortic dissection”, is the core research cluster of endovascular interventional therapy for type B aortic dissection; the yellow cluster, represented by “aortic surgery”, focuses on the technical optimization of aortic dissection surgery and the prevention and control of neurological complications; the blue cluster focuses on the research of risk factors, prognosis evaluation and risk prediction model construction of aortic dissection, with representative keywords “mortality” and “outcome”; the orange cluster includes keywords such as “inflammation” and “vascular smooth muscle cell”, corresponding to the research on pathogenesis, vascular biomechanics and basic medicine of aortic dissection. The four-quadrant diagram analysis of representative keywords is shown in Figure 9B: the driving themes in the upper right quadrant, centered on “aortic dissection” and “aorta”, have high centrality and high development degree, and are the core research hotspots and development driving forces of the current field; the basic themes in the lower right quadrant include “mortality”, “type b aortic dissection”, etc., which are classic basic themes of the entire field; the upper left quadrant presents niche themes with high specialization but weak cross-domain relevance; the lower left quadrant represents themes currently on the edge of the field, which may be emerging frontier directions or research topics that were prevalent but have now declined.

The keyword time trend evolution diagram is shown in Figure 9C and Supplementary Figure S2A, combined with the variable-importance-in-projection (VIP) score diagram (Supplementary Figure S2C): “aortic dissection” and “acute aortic dissection” have been firmly in the forefront throughout the time span from 2010 to 2025, and are stable core themes in the field; from 2010 to 2015, the research focused on open surgery and early endovascular therapy; from 2016 to 2020, the focus shifted to precise classification treatment and surgical optimization of type A/B aortic dissection; while terms such as “type a aortic dissection”, “type b aortic dissection”, “case report” and “risk factor” have increased in popularity in the past five years, representing the core research hotspots in the field in recent years, reflecting that the research focus of the field has gradually shifted from open surgical treatment to endovascular interventional therapy, precise and intelligent prognosis prediction, and basic mechanism exploration.

The keyword similarity clustering heat map (Supplementary Figure S2B) shows the popularity correlation of keywords, and clusters keywords with high popularity in similar time periods into four clusters marked with different colors: the yellow module includes the research direction of endovascular interventional therapy; the purple module revolves around the research direction of prognosis evaluation and risk prediction; the light blue module corresponds to the research direction of imaging diagnosis and evaluation; the orange module focuses on the research direction of disease classification and surgical treatment. The conceptual structure diagram of multiple correspondence analysis (MCA) (Supplementary Figure S2D) clearly presents the conceptual structure and clustering association of keywords in the field, forming three core conceptual clusters: the red cluster (“tevar”, “type b aortic dissection”, “stent graft”, etc.), focusing on endovascular interventional therapy and postoperative vascular remodeling of type B aortic dissection; the purple cluster (“type a aortic dissection”, “frozen elephant trunk”, etc.), revolving around surgical treatment, disease diagnosis and basic phenotype research of type A aortic dissection; the green cluster (“machine learning”, “nomogram”, “prediction model”), focusing on prognostic risk prediction, complication evaluation and artificial intelligence model construction of aortic dissection.

### 3.7 Highly cited reference analysis

The citation frequency of a paper can indirectly reflect its academic quality to a certain extent, and intuitively show its attention and academic influence in the corresponding field. In-depth analysis of highly cited papers enables researchers to efficiently and comprehensively grasp the core research hotspots in the field (Table 6). The most concerned article in the field of aortic dissection research is *Presentation, Diagnosis, and Outcomes of Acute Aortic Dissection: 17-Year Trends From the International Registry of Acute Aortic Dissection* by Pape et al., published in the *Journal of the American College of Cardiology* in 2015, which studied the changing trends of clinical manifestations, diagnosis and treatment outcomes of acute aortic dissection (AAD) over 17 years. The results showed that although the clinical manifestations of AAD did not change significantly, the use of computed tomography (CT) examination increased, surgical treatment was more common in type A AAD, while endovascular treatment for type B AAD increased. The in-hospital mortality of type A AAD was significantly reduced, but there was no significant change in type B AAD (18). In addition, articles by Nienaber et al. (19) and Mészáros et al. (20) also provided great reference value for subsequent research.

**Table 6:** Ranking of the top fifteen major highly cited references in the field of aortic dissection from 2000 to 2025.

Rank	Author	Article title	Source title	Cited	Year	Document type	DOI
1	Pape, LA; Awais, M; Woznicki, EM et al.	Presentation, Diagnosis, and Outcomes of Acute Aortic Dissection 17-Year Trends From the International Registry of Acute Aortic Dissection	Journal of the American College of Cardiology	886	2015	Article	10.1016/j.jacc.2015.05.029
2	Nienaber, CA; Kische, S; Rousseau, H et al.	Endovascular Repair of Type B Aortic Dissection Long-term Results of the Randomized Investigation of Stent Grafts in Aortic Dissection Trial	Circulation-Cardiovascular Interventions	859	2013	Article	10.1161/CIRCINTERVENTIONS.113.000463
3	Mészáros, I; Mórocz, J; Szlávi, J et al.	Epidemiology and clinicopathology of aortic dissection - A population-based longitudinal study over 27 years	Chest	754	2000	Article	10.1378/chest.117.5.1271
4	Howard, DPJ; Banerjee, A; Fairhead, JF et al.	Population-Based Study of Incidence and Outcome of Acute Aortic Dissection and Premorbid Risk Factor Control 10-Year Results From the Oxford Vascular Study	Circulation	612	2013	Article	10.1161/CIRCULATIONAHA.112.000483
5	Nienaber, CA; Rousseau, H; Eggebrecht, H et al.	Randomized Comparison of Strategies for Type B Aortic Dissection The INvestigation of STEnt Grafts in Aortic Dissection (INSTEAD) Trial	Circulation	608	2009	Article	10.1161/CIRCULATIONAHA.109.886408
6	Lombardi, JV; Hughes, GC; Appoo, JJ et al.	Society for Vascular Surgery (SVS) and Society of Thoracic Surgeons (STS) reporting standards for type B aortic dissections	Journal of Vascular Surgery	477	2020	Article	10.1016/j.jvs.2019.11.013
7	Suzuki, T; Mehta, RH; Ince, H et al.	Clinical profiles and outcomes of acute type B aortic dissection in the current era: Lessons from the International Registry of Aortic Dissection (IRAD)	Circulation	409	2003	Article	10.1161/01.cir.0000087386.07204.09
8	Khan, IA; Nair, CK	Clinical, diagnostic, and management perspectives of aortic dissection	Chest	407	2002	Review	10.1378/chest.122.1.311
9	Tsai, TT; Trimarchi, S; Nienaber, CA	Acute Aortic Dissection: Perspectives from the International Registry of Acute Aortic Dissection (IRAD)	European Journal of Vascular and Endovascular Surgery	347	2009	Article	10.1016/j.ejvs.2008.11.032
10	Malaisrie, SC; Szeto, WY; Halas, M et al.	2021 The American Association for Thoracic Surgery expert consensus document: Surgical treatment of acute type A aortic dissection	Journal of Thoracic and Cardiovascular Surgery	250	2021	Article; Proceedings Paper	10.1016/j.jtcvs.2021.04.053
11	Nienaber, CA; Kische, S; Zeller, T et al.	Provisional extension to induce complete attachment after stent-graft placement in type B aortic dissection:: The PETTICOAT concept	Journal of Endovascular Therapy	245	2006	Article	10.1583/06-1923.1
12	Dong, ZH; Fu, WG; Wang, YQ et al.	Retrograde Type A Aortic Dissection After Endovascular Stent Graft Placement for Treatment of Type B Dissection	Circulation	236	2009	Article	10.1161/CIRCULATIONAHA.107.759076
13	Rogers, AM; Hermann, LK; Booher, AM et al.	Sensitivity of the Aortic Dissection Detection Risk Score, a Novel Guideline-Based Tool for Identification of Acute Aortic Dissection at Initial Presentation Results From the International Registry of Acute Aortic Dissection	Circulation	231	2011	Article	10.1161/CIRCULATIONAHA.110.988568
14	Zhu, YJ; Lingala, B; Baiocchi, M et al.	Type A Aortic Dissection-Experience Over 5 Decades JACC Historical Breakthroughs in Perspective	Journal of the American College of Cardiology	219	2020	Review	10.1016/j.jacc.2020.07.061
15	Kurihara, T; Shimizu-Hirota, R; Shimoda, M et al.	Neutrophil-Derived Matrix Metalloproteinase 9 Triggers Acute Aortic Dissection	Circulation	217	2012	Article	10.1161/CIRCULATIONAHA.112.097097

**Figure 10:**
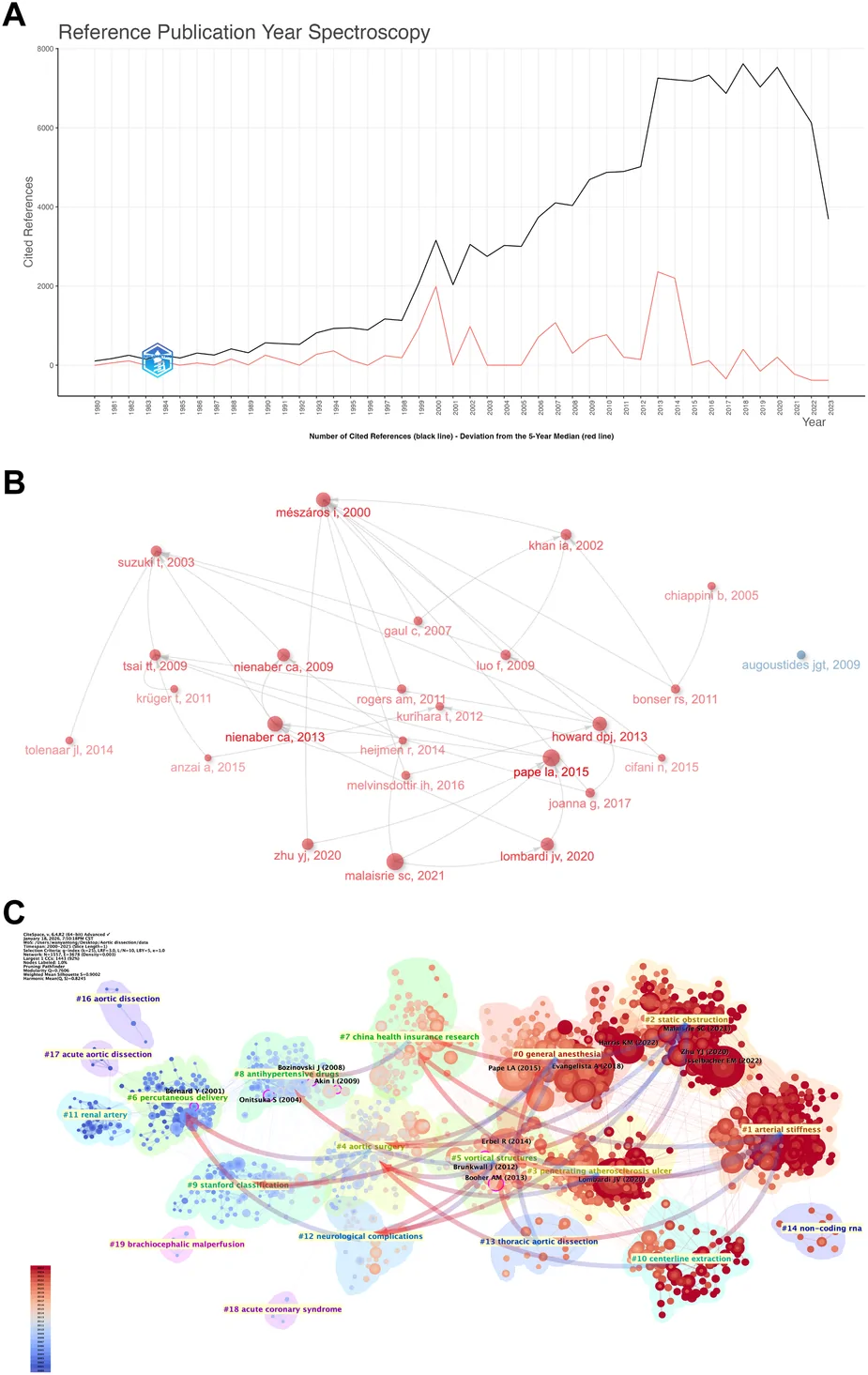
**(A)** RPYS plot, where the black curve shows the annual changes in the number of cited references in the field of aortic dissection research from 1960 to 2023, and the red curve presents its deviation from the 5-year median, identifying the key time nodes and development stages of knowledge accumulation in this field. **(B)** Co-citation network map of highly cited references, where the node size corresponds to the co-citation frequency of each reference, and lines represent the co-citation association between literatures, clearly presenting the knowledge association relationships between foundational and landmark studies in this field. **(C)** Theme clustering map of highly cited references, which visualizes the theme clustering characteristics of highly cited literatures in the field through clustering, identifies a total of 20 core research theme clusters, and reveals the evolution context and knowledge structure of research topics in this field.

The Reference Publication Year Spectroscopy (RPYS) in Figure 10A shows that 1990–1999 was the knowledge accumulation period of the field, with the total number of cited references at a low level; the first cited peak appeared in 2000, and the core research system was initially formed; after 2010, the total number of citations continued to rise, reaching the peak from 2018 to 2020, which was the concentrated outbreak period of research results in the field; after 2021, it showed a downward trend, which simultaneously located the key nodes of the academic development of the field. Figure 10B shows the co-citation network diagram of highly cited research in the field, with arrows pointing from citing papers to cited references, and nodes of different colors representing different research directions. Among them, early studies such as Mészáros, I (2000) (20) and Khan, IA (2002) (21) are the foundational core literatures of this academic context; literatures such as Pape, LA (2015) (18), Nienaber, CA (2013) (19), and Howard, DPJ (2013) (22) played a connecting role between the preceding and the following; Augoustides, JGT (2009) (23) formed a relatively independent research branch, expanding the research dimension of the field.

Figure 10C intuitively presents the potential association between these highly cited studies through clustering visualization technology, and a total of 20 core research theme clusters have been identified. #0 general anesthesia is the largest core cluster, and the literatures marked with purple circles are the key hub nodes of each theme. Cross-cluster associations reveal the evolutionary law of the field's research from classic disease classification and surgical treatment to cross-cutting expansion of perioperative management, minimally invasive intervention, and complication prevention and control.

### 3.8 Single-Cell transcriptome profiling and independent validation

**Figure 11:**
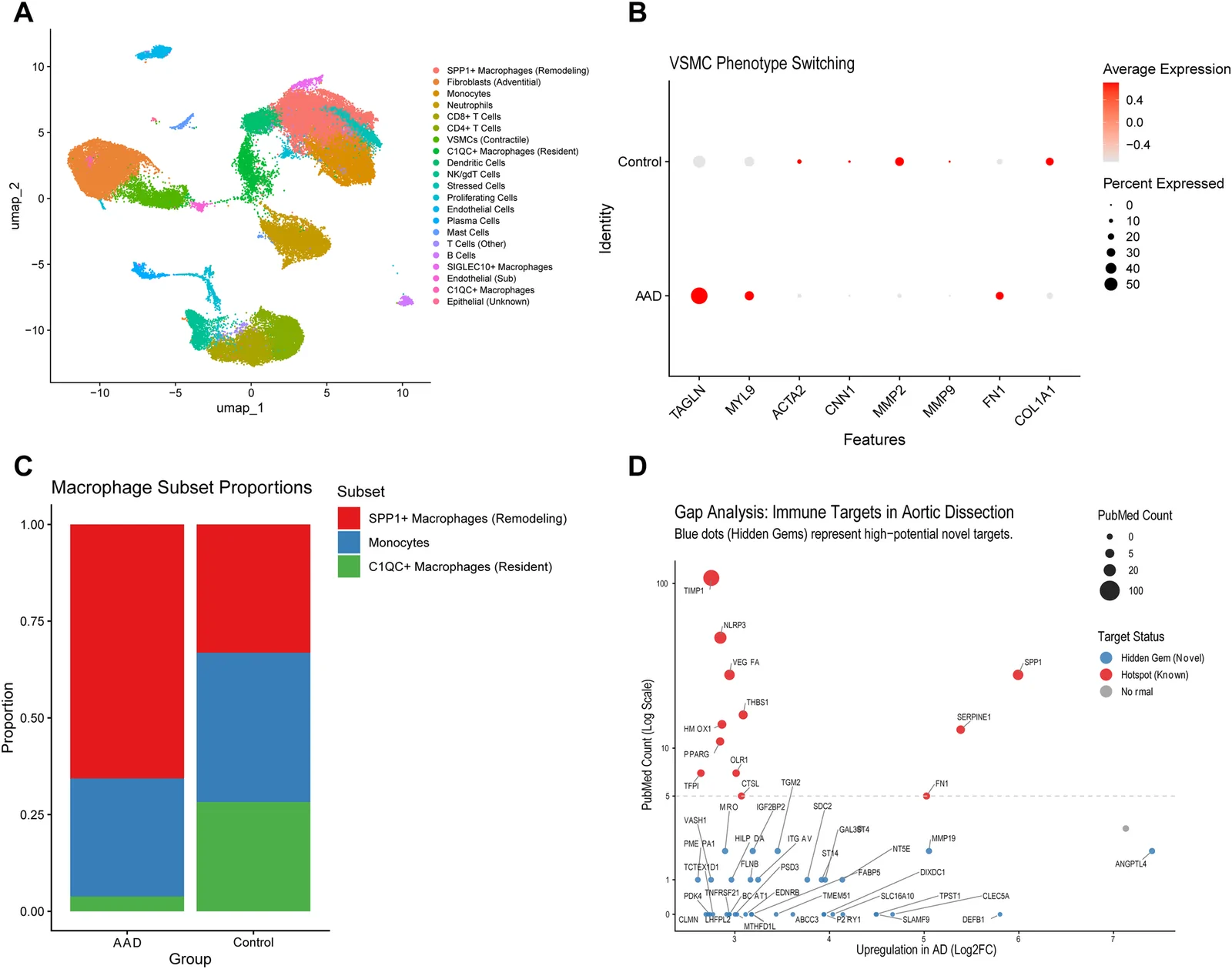
Single-Cell transcriptomic analysis of the discovery cohort and target-Gap screening. **(A)** UMAP of major cell populations in GSE254132. **(B)** Dot plot of VSMC contractile and extracellular-matrix markers in AD and control tissue; dot size denotes the percentage of expressing cells and color denotes average expression. **(C)** Group-level proportions of SPP1⁺ remodeling macrophages, monocytes, and C1QC⁺ resident macrophages. **(D)** Exploratory macrophage target-gap plot relating donor-pseudobulk up-regulation to AD-related PubMed counts. Blue points denote literature-sparse candidates, not proven novel or therapeutic targets; red points denote established or active topics.

Analysis of GSE254132 resolved the principal structural and immune compartments of AD and non-dissected aortic tissue, including contractile VSMCs, adventitial fibroblasts, endothelial cells, monocytes/macrophages, T lymphocytes, dendritic cells, and neutrophils (Figure 11A). The separation of these lineages provided the cellular framework for testing the bibliometrically identified themes of VSMC remodeling and immune-microenvironment change.

Within the discovery cohort, AD VSMCs showed higher *TAGLN* and *MYL9* expression together with increased *FN1*, whereas *ACTA2*, *CNN1*, *MMP2*, *MMP9*, and *COL1A1* were higher in controls (Figure 11B). The coexistence of retained contractile markers and enhanced *FN1* indicates a heterogeneous remodeling program rather than a uniform binary conversion from a contractile to a synthetic state.

Macrophage-state analysis in GSE254132 showed a larger group-level proportion of SPP1⁺ remodeling macrophages in AD, whereas C1QC⁺ resident macrophages and monocytes accounted for a greater share of macrophages in controls (Figure 11C). Because this pattern has been reported in prior AD studies, it was interpreted as biological concordance with the bibliometric inflammation theme rather than as a novel macrophage phenotype.

Independent reanalysis of GSE213740 identified the expected vascular and immune lineages (Supplementary Figure S3A). Compared with controls, AD VSMCs had lower *TAGLN*, *MYL9*, *ACTA2*, and *CNN1* and higher *MMP2*, *MMP9*, and *FN1*, reproducing the direction of contractile-program loss and extracellular-matrix remodeling at the program level (Supplementary Figure S3B). SPP1⁺ remodeling macrophages were modestly more abundant in AD, but C1QC⁺ resident macrophages remained the predominant state in both groups (Supplementary Figure S3C). Thus, the external cohort supported the direction of VSMC and macrophage remodeling but not the magnitude or dominance observed in GSE254132. Its donor-pseudobulk target ranking also differed and did not independently nominate *ANGPTL4* or *MMP19* as leading macrophage candidates (Supplementary Figure S3D).

### 3.9 Bibliometric–single-cell joint analysis of candidate targets

The joint analysis used differential expression to define biologically prioritized genes and target-level publication data to determine whether those genes represented mature AD topics or literature gaps. Established AD-associated genes, including *TIMP1*, *NLRP3*, *VEGFA*, and *SPP1*, occupied the high-publication portion of the discovery-cohort gap plot, whereas *ANGPTL4* and *MMP19* combined marked expression differences with little AD-specific literature (Figure 11D). This classification denotes a research opportunity, not proof of novelty, causality, or druggability.

**Figure 12:**
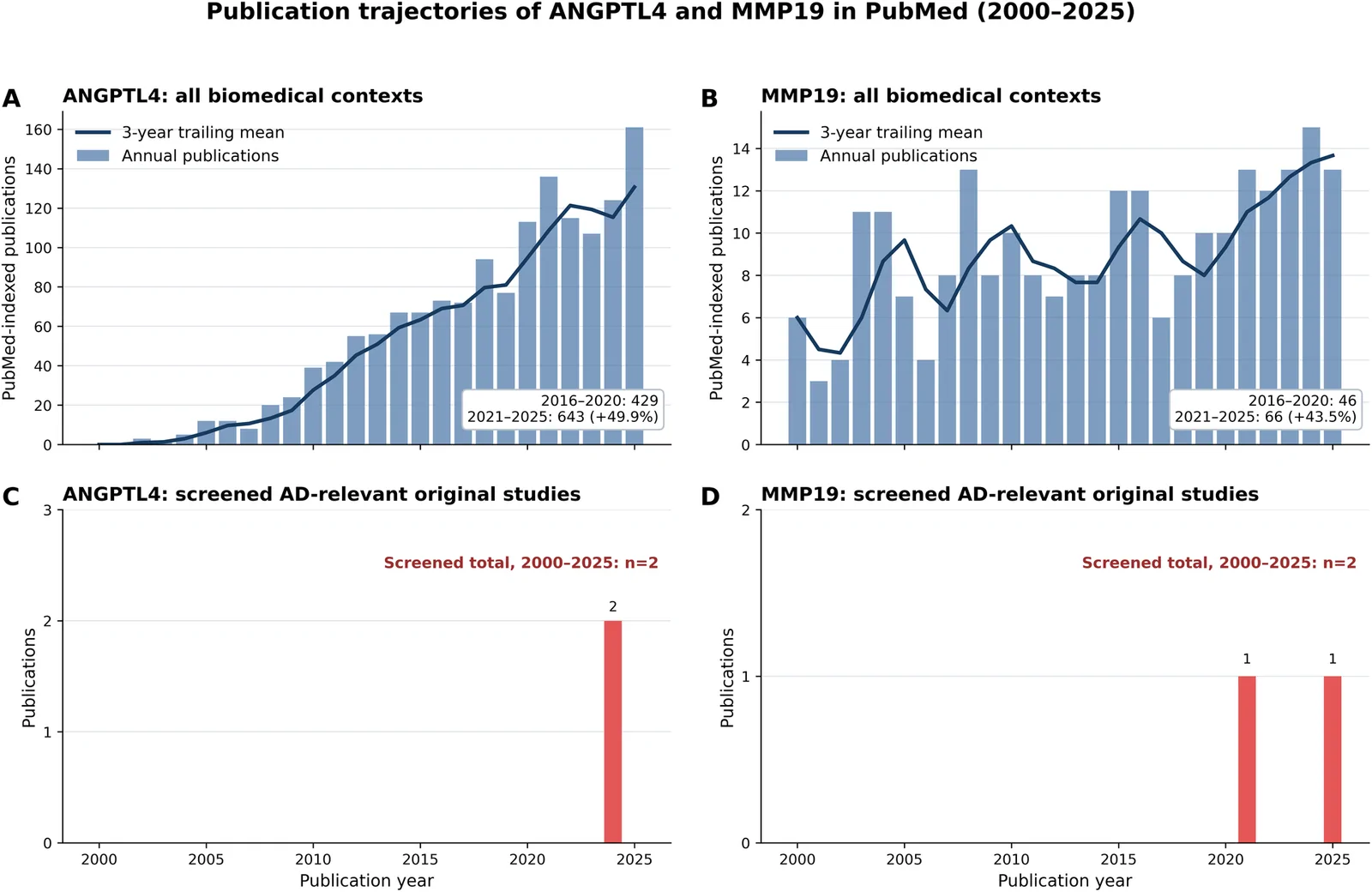
Publication trends of *ANGPTL4* and *MMP19* in pubMed, 2000–2025. **(A,B)** Annual title/abstract-indexed publications in all biomedical contexts; bars show annual counts and lines show the three-year trailing mean. Each PMID was counted once using the journal issue year. **(C,D)** Screened original AD-relevant publications returned by the author-specified unfielded Boolean searches. From 2016 to 2020 to 2021–2025, *ANGPTL4* publications increased from 429 to 643 (+49.9%) and *MMP19* publications from 46 to 66 (+43.5%). Two original AD-relevant publications were retained for each target; non-target *ANGPTL8* records and the *MMP19* corrigendum were excluded. Searches were retrieved on July 19, 2026, and journal issue years were restricted through 2025. The figure distinguishes rising general interest from sparse AD-specific evidence.

The standardized PubMed trend analysis provided a more direct test of this interpretation. *ANGPTL4* appeared in 1,483 title/abstract-indexed publications during 2000–2025; publications increased from 429 in 2016–2020 to 643 in 2021–2025 (+49.9%). *MMP19* appeared in 240 publications, increasing from 46 to 66 between the same five-year periods (+43.5%). After the issue-year restriction and PMID-level screening of the author-specified AD searches, two original *ANGPTL4*-related publications were retained, both from 2024, and two original *MMP19*-related publications were retained, from 2021 to 2025. The 2025 *MMP19* corrigendum was not counted as a separate study. The two targets therefore show rising interest across biomedicine but remain sparsely examined in AD (Figure 12).

## 4 Discussion

### 4.1 Principal findings and integrative rationale

Three findings organize the interpretation of this study. First, AD research has moved from low-volume output to sustained expansion, but publication volume, citation influence, and collaboration centrality are not distributed in the same way: China contributed the largest corpus, while the United States retained the greatest citation impact and international link strength. Second, independent keyword, co-citation, and temporal analyses converged on four fronts—endovascular treatment, vascular-wall inflammation and remodeling, molecular candidate discovery, and data-driven prognosis—making these themes less dependent on any single mapping method. Third, single-cell analysis was added as a mechanistic cross-check and gene-prioritization layer, not to re-label established observations such as VSMC plasticity or SPP1⁺ macrophages as first discoveries. Bibliometrics identified mature and emerging questions; transcriptomics tested whether the corresponding biological programs were detectable in human tissue; and target-level publication trends separated well-developed genes from expression-supported but literature-sparse candidates. This linkage generates testable hypotheses but does not establish causality or therapeutic validity.

### 4.2 Interpretation of major research frontiers

The four frontiers were retained only when supported by more than one bibliometric signal, such as keyword growth, thematic position, or co-citation structure. Their interpretation below therefore emphasizes what the present maps add to existing evidence and where the evidence remains incomplete.

#### 4.2.1 From open surgery to endovascular therapy: a shift in the research question

The rising frequency and centrality of TEVAR-related terms, together with the declining relative prominence of conventional surgery terms, indicate that the principal type B AD question has shifted from whether endovascular repair is feasible to which patients, devices, and anatomical settings derive durable benefit. Registry and comparative evidence supports increasing TEVAR use and improved short-term outcomes in complicated type B disease (24–27). The maps should not be read as evidence that open surgery is becoming irrelevant: type A AD remains predominantly surgical, and endovascular arch or root strategies are still limited to selected anatomy and specialist centers (28–32). For uncomplicated type B AD, the evidence base remains less definitive and careful indication control is still required (33, 34).

The associated growth of imaging, false-lumen, remodeling, and complication keywords further shows that procedural success is no longer defined only by technical deployment. Tear location, branch-vessel perfusion, false-lumen patency, and longitudinal aortic remodeling increasingly determine device selection and surveillance (35–37). The research opportunity is therefore comparative and longitudinal: multicenter studies should connect standardized imaging phenotypes with device configuration, neurological complications, reintervention, and late aortic events. This is also where the collaboration network matters, because the relatively small number of complex cases at any one center limits robust subgroup analysis.

#### 4.2.2 Inflammation, VSMC plasticity, and macrophage-state heterogeneity

Inflammation and VSMC-related terms occupied connected, increasingly visible positions in the maps, consistent with a model in which immune-cell recruitment, extracellular-matrix degradation, and VSMC state change reinforce one another. Studies of macrophage recruitment and inflammatory signaling support the immune component (38–40). VSMC plasticity and degenerative transcriptional or epigenetic programs have been reported in AD and related aortic disease (41–45), while single-cell and mechanistic studies further connect VSMC states to extracellular-matrix remodeling (46, 47). The discovery single-cell cohort supported this connection but also showed that phenotype switching is not adequately described as a simple loss of every contractile marker: *TAGLN* and *MYL9* remained high in AD VSMCs while *FN1* increased. In the independent GSE213740 cohort, the more conventional pattern of reduced contractile markers and increased *MMP2*, *MMP9*, and *FN1* was observed. The shared signal is therefore a remodeling program, whereas the individual marker pattern is cohort-dependent.

Macrophage results require the same restraint. GSE254132 showed a marked expansion of SPP1⁺ remodeling macrophages, while GSE213740 showed only a modest increase and retained C1QC⁺ resident macrophages as the largest state. This heterogeneity may reflect tissue sampling, disease stage, donor characteristics, dissociation, or state-label definitions. It does not negate a role for remodeling macrophages, which is supported by prior studies (40, 48–50), but it argues against presenting their expansion as universally dominant. The contribution of the present analysis is the donor-aware cross-cohort comparison and its alignment with the bibliometric inflammation theme, not the first description of SPP1-associated macrophages.

#### 4.2.3 ANGPTL4 and MMP19: literature-sparse candidates, not validated therapies

The target-gap analysis behaved as expected for established AD biology: *TIMP1*, *NLRP3*, *VEGFA*, *SPP1*, *MMP8*, and *S100A8/A9* combined disease-relevant expression with a substantial literature base (51, 52). *ANGPTL4* and *MMP19* occupied a different position. Their broader PubMed output increased by 49.9% and 43.5%, respectively, between the two most recent five-year periods, demonstrating rising biomedical interest, yet only two original AD-relevant publications were identified for each target through 2025. This combination—not expression alone and not a small static PubMed count—is the basis for prioritizing them for further study (Figure 12).

The biological support remains unequal. *ANGPTL4* has direct, although still limited, AD evidence: a 2024 study reported that *ANGPTL4* promoted human aortic smooth muscle cell phenotypic switching and dysfunction through phosphoinositide 3-kinase/protein kinase B (PI3 K/AKT) signaling (53). Its expression signal and rapidly expanding literature justify replication and mechanistic testing, but neither establishes clinical utility. For *MMP19*, a 2021 study linked *MMP19* to a matrix-degradation-associated long noncoding RNA network without direct perturbation (54). A 2025 study subsequently provided direct preclinical evidence: systemic or vascular smooth muscle cell-specific *Mmp19* loss worsened *β*-aminopropionitrile-induced thoracic aortic aneurysm and dissection, whereas vascular smooth muscle cell-specific *MMP19* complementation alleviated disease through the *MMP19*/Aggrecan/Wnt/*β*-catenin axis (55). This functional evidence strengthens the mechanistic rationale but remains based largely on experimental models; it does not establish *MMP19* as a clinically validated therapeutic target.

A credible translational sequence would now require independent replication of the reported *MMP19*/Aggrecan/Wnt/*β*-catenin mechanism, spatial or immunohistochemical localization in independent human AD tissue, confirmation at the protein level, and perturbation experiments in human VSMC, endothelial-cell, and macrophage systems. Because the published functional evidence derives principally from a *β*-aminopropionitrile-induced mouse model, additional *in vivo* models should test reproducibility, treatment timing, safety, and possible off-target effects on extracellular-matrix homeostasis. Until these steps are completed, the present results support resource prioritization and hypothesis generation rather than clinical drug nomination.

#### 4.2.4 Precision prognosis and machine learning: growth with a validation gap

The recent rise of “machine learning”, “nomogram”, and related keywords marks a genuine methodological shift from isolated risk factors toward multivariable prediction. Published models address perioperative mortality, organ malperfusion, and rupture (56–59); respiratory, renal, bleeding, and diagnostic outcomes (60–63); and longer-term mortality or intensive care unit (ICU) outcomes (64–67), often reporting good internal discrimination. However, high reported area-under-the-curve values do not by themselves demonstrate transportability. Many studies remain retrospective, single-center, and vulnerable to small event counts, data leakage, variable outcome definitions, and incomplete calibration assessment. The bibliometric growth signal therefore identifies an active frontier, but the clinically important question is now external and prospective validation rather than production of additional single-center models.

The next stage should prioritize common data definitions, temporal validation, multicenter calibration, and comparison with existing clinical scores. Imaging segmentation and radiomic features may improve prediction (68–70), and molecular or multi-omic biomarkers have also been proposed (71–75); however, their incremental value should be reported beyond standard clinical variables and balanced against acquisition cost and missingness. Interpretable methods such as Shapley additive explanations (SHAP) can describe model behavior but cannot correct biased training data (76). Registry-based collaboration, particularly across the geographically separated clusters identified here, offers a practical route to sufficiently diverse validation cohorts.

### 4.3 Strengths and limitations

A principal strength is triangulation across two bibliographic databases, several mapping methods, a growth model, donor-aware single-cell analysis, and an independent single-cell cohort. WoSCC contributed standardized citation and cited-reference metadata, while Scopus broadened disciplinary and source coverage; deduplication reduced overlap. The target-level PubMed analysis added a transparent biomedical search that directly tested whether ANGPTL4 and MMP19 were simultaneously attracting broader attention and remaining underrepresented in AD. Importantly, the external cohort was used to evaluate program-level reproducibility rather than requiring identical cluster labels or effect sizes.

Several limitations affect interpretation. WoSCC and Scopus have overlapping but non-identical journal, regional, conference, and citation coverage; combining them reduces but does not remove database bias. Restricting the primary corpus to English-language, title-and-keyword-matched records may underrepresent non-English research and studies in which AD appears only in the abstract. Database update timing, author and institution disambiguation, and the shorter citation window for recent papers can alter rankings and network centrality. Publication counts and citations indicate attention, not methodological quality, and logistic forecasts are sensitive to indexing completeness and model form. PubMed results remain sensitive to automatic term mapping, phrase selection, publication type, and multiple electronic and print dates; PMID-level screening and issue-year assignment reduced but did not eliminate this source of subjectivity. The searches may also miss historical synonyms or full-text-only mentions. The single-cell cohorts remain small and cross-sectional, and differences in tissue source, disease stage, and processing probably contributed to the unequal macrophage effect sizes. GSE213740 reproduced broad VSMC and macrophage programs but did not independently prioritize *ANGPTL4* or *MMP19*. Neither candidate was validated at the protein or functional level in the present cohorts. Although a 2025 experimental study provides direct mechanistic support for a protective role of *MMP19*, its clinical relevance and therapeutic safety remain unknown.

## 5 Conclusions

This study characterizes the global development, collaboration structure, and thematic evolution of AD research from 2000 to 2025 using complementary WoSCC and Scopus records. Publication output expanded rapidly after 2010, while country-level analysis showed a distinction between volume leadership in China and citation and collaboration leadership in the United States. The maps converged on four priorities: refinement of endovascular treatment and long-term remodeling surveillance, inflammatory and VSMC mechanisms, molecular candidate discovery, and externally validated prediction models. These areas should be interpreted as connected research programs rather than independent trends, because procedural outcomes, vascular-wall biology, biomarkers, and risk prediction increasingly rely on shared multicenter and multimodal data. The single-cell component was used to test mechanistic themes and prioritize genes, not to claim first discovery of VSMC plasticity or SPP1⁺ macrophages. GSE213740 reproduced VSMC remodeling and the direction of SPP1⁺ macrophage change but showed a smaller macrophage effect, underscoring biological and technical heterogeneity. Target-level PubMed trends showed that ANGPTL4 and MMP19 are attracting broader biomedical interest while remaining sparsely examined in AD. Accordingly, *ANGPTL4* is a plausible candidate for replication and functional testing, whereas *MMP19* has emerging direct preclinical mechanistic support and should be regarded as an unvalidated translational candidate rather than an established therapy. Neither molecule should yet be presented as a clinically validated therapeutic target. Spatial, protein-level, perturbational, and *in vivo* validation will be required to determine whether either candidate can be translated safely to AD.

Future work should combine multicenter clinical registries with standardized imaging, longitudinal outcomes, and experimentally validated molecular measurements. Such designs would address the two main gaps identified here: insufficient external validation of prediction models and insufficient functional validation of literature-sparse molecular candidates.

## Data Availability

The raw data supporting the conclusions of this article will be made available by the authors, without undue reservation.
