# Supplementary material for: Bridging macro bibliometrics and micro molecular mechanisms: an integrated analysis of global aortic dissection research
Source: Front Cardiovasc Med. 2026 Aug 31;13:1822129. doi: 10.3389/fcvm.2026.1822129 (PMC13572280; doi:10.3389/fcvm.2026.1822129)
Supplement: Supplementary file 1 [file Datasheet1.docx]

**Supplementary Figure 1**

Global and Corpus-Specific Bibliometric Profile of Christoph A. Nienaber. (A) OpenAlex indicators, annual output, and major research topics. (B) Metrics calculated within the AD corpus, including local publications, citations, h-index, annual output, and keywords.

**Supplementary Figure 2**

Supplementary Keyword Analyses. (A) Circular heat map of standardized annual keyword frequency from 2016 to 2025. (B) Hierarchically clustered keyword-similarity heat map. (C) VIP scores for recent keywords. (D) MCA showing the conceptual structure and proximity of keyword themes.

**Supplementary Figure 3**

Independent Single-Cell Validation in GSE213740. (A) Harmony-integrated UMAP of vascular and immune cell states from six acute type A aortic dissection donors and three controls. (B) VSMC marker dot plot; color denotes average expression and dot size denotes the percentage of expressing cells. AD VSMCs showed lower *TAGLN*, *MYL9*, *ACTA2*, and *CNN1* and higher *MMP2*, *MMP9*, and *FN1*. (C) Group-level proportions of SPP1⁺ remodeling macrophages, monocytes, and C1QC⁺ resident macrophages; the SPP1⁺ fraction was modestly higher in AD, while C1QC⁺ macrophages remained predominant. (D) Donor-pseudobulk target-gap plot relating AD up-regulation to PubMed count; colors denote literature class and point size denotes publication count. This cohort supports program-level VSMC and macrophage remodeling but does not independently validate ANGPTL4 or MMP19 as leading candidates.
